# A Mobile Health Solution Complementing Psychopharmacology-Supported Smoking Cessation: Randomized Controlled Trial

**DOI:** 10.2196/17530

**Published:** 2020-04-27

**Authors:** Laura Carrasco-Hernandez, Francisco Jódar-Sánchez, Francisco Núñez-Benjumea, Jesús Moreno Conde, Marco Mesa González, Antón Civit-Balcells, Santiago Hors-Fraile, Carlos Luis Parra-Calderón, Panagiotis D Bamidis, Francisco Ortega-Ruiz

**Affiliations:** 1 Smoking Cessation Unit Medical-Surgical Unit of Respiratory Diseases Virgen del Rocío University Hospital Seville Spain; 2 Centro de Investigación Biomédica en Red de Enfermedades Respiratorias Carlos III Institute of Health Madrid Spain; 3 Research and Innovation Group in Biomedical Informatics Biomedical Engineering and Health Economy, Institute of Biomedicine of Seville Virgen del Rocío University Hospital, Spanish National Research Council, University of Seville Seville Spain; 4 Department of Architecture and Computer Technology School of Computer Engineering Universidad de Sevilla Seville Spain; 5 Salumedia Labs Seville Spain; 6 Medical Physics Laboratory School of Medicine Aristotle University of Thessaloniki Thessaloniki Greece

**Keywords:** smoking cessation, behavioral change, health recommender systems, mHealth, randomized controlled trial

## Abstract

**Background:**

Smoking cessation is a persistent leading public health challenge. Mobile health (mHealth) solutions are emerging to improve smoking cessation treatments. Previous approaches have proposed supporting cessation with tailored motivational messages. Some managed to provide short-term improvements in smoking cessation. Yet, these approaches were either static in terms of personalization or human-based nonscalable solutions. Additionally, long-term effects were neither presented nor assessed in combination with existing psychopharmacological therapies.

**Objective:**

This study aimed to analyze the long-term efficacy of a mobile app supporting psychopharmacological therapy for smoking cessation and complementarily assess the involved innovative technology.

**Methods:**

A 12-month, randomized, open-label, parallel-group trial comparing smoking cessation rates was performed at Virgen del Rocío University Hospital in Seville (Spain). Smokers were randomly allocated to a control group (CG) receiving usual care (psychopharmacological treatment, n=120) or an intervention group (IG) receiving psychopharmacological treatment and using a mobile app providing artificial intelligence–generated and tailored smoking cessation support messages (n=120). The secondary objectives were to analyze health-related quality of life and monitor healthy lifestyle and physical exercise habits. Safety was assessed according to the presence of adverse events related to the pharmacological therapy. Per-protocol and intention-to-treat analyses were performed. Incomplete data and multinomial regression analyses were performed to assess the variables influencing participant cessation probability. The technical solution was assessed according to the precision of the tailored motivational smoking cessation messages and user engagement. Cessation and no cessation subgroups were compared using t tests. A voluntary satisfaction questionnaire was administered at the end of the intervention to all participants who completed the trial.

**Results:**

In the IG, abstinence was 2.75 times higher (adjusted OR 3.45, *P*=.01) in the per-protocol analysis and 2.15 times higher (adjusted OR 3.13, *P*=.002) in the intention-to-treat analysis. Lost data analysis and multinomial logistic models showed different patterns in participants who dropped out. Regarding safety, 14 of 120 (11.7%) IG participants and 13 of 120 (10.8%) CG participants had 19 and 23 adverse events, respectively (*P*=.84). None of the clinical secondary objective measures showed relevant differences between the groups. The system was able to learn and tailor messages for improved effectiveness in supporting smoking cessation but was unable to reduce the time between a message being sent and opened. In either case, there was no relevant difference between the cessation and no cessation subgroups. However, a significant difference was found in system engagement at 6 months (*P*=.04) but not in all subsequent months. High system appreciation was reported at the end of the study.

**Conclusions:**

The proposed mHealth solution complementing psychopharmacological therapy showed greater efficacy for achieving 1-year tobacco abstinence as compared with psychopharmacological therapy alone. It provides a basis for artificial intelligence–based future approaches.

**Trial Registration:**

ClinicalTrials.gov NCT03553173; https://clinicaltrials.gov/ct2/show/NCT03553173

**International Registered Report Identifier (IRRID):**

RR2-10.2196/12464

## Introduction

Tobacco use presents a major preventable public health problem; it is the leading cause of health deterioration and premature death. The World Health Organization recognizes smoking as a chronic systemic disease among the addictions capable of producing high physical damage related to multiple diseases [1]. Further, smoking kills over 7 million people annually, with global costs estimated at US $1.4 trillion [2].

Validated approaches to facilitate smoking cessation include nicotine replacement therapy, pharmacological treatment (ie, bupropion and varenicline), and behavioral and psychological support. Combining behavioral and psychological support with pharmacological treatment is currently the most effective intervention for achieving tobacco abstinence [3]. Moreover, bupropion was shown to approximately double the likelihood of long-term tobacco abstinence as compared with placebo [4]. Furthermore, varenicline (2 mg, total daily dose) was shown to triple the likelihood of maintaining long-term tobacco abstinence as compared with placebo [5]. Despite the proven efficacy of these treatments, a meaningful number of smokers fail to stop smoking, and many factors influence their success, including the motivation to stop and continuous support.

Mobile and web-based interventions have been used to facilitate effective behavioral changes in different health domains [6-9], including smoking cessation. Scientific evidence has indicated that mobile phone–based text messages supporting smoking cessation were nearly 1.7 times more successful than a control approach at 6 months [10]. It has been proven that the behavioral change impact is higher when users receive multiple tailored health recommendations [11,12]. However, in previous studies, a traditional computer-tailoring approach was used to generate such personalized health recommendations. Computer tailoring involves the generation of patient-specific recommendations, typically in the form of messages, by computers, and it is performed after personal assessment to match the characteristics, needs, and interests of the patients [13-16]. Cupertino et al [17] recently piloted a 12-week smoking cessation program involving text messaging with pharmacotherapy support, which showed promising abstinence rates at 3 months and high participant satisfaction. However, there is no evidence for the long-term abstinence efficacy of mobile-based tailored interventions combined with psychopharmacological therapies.

As part of the SmokeFreeBrain H2020 European Commission project [18], the Social-Local-Mobile (So-Lo-Mo) study investigated mobile and artificial intelligence (AI) technologies (health recommender system [HRS]) as a complementary aid to pharmacological treatments. The experimental intervention focused on providing ubiquitous tailored support to patients willing to stop smoking through a digital therapeutic mobile app solution.

Participants receiving the So-Lo-Mo intervention were provided with access to a code to be introduced in the app. The code activated the app, connecting it with the hospital patient database. The app automatically downloaded the necessary data to initialize the app profile, reducing the burden of having to create a user profile. The mobile app was connected with an AI system designed to learn from patient interests through their interactions with the app to dynamically (1) determine, personalize, and send motivational messages to support smoking cessation; (2) schedule the message delivery frequency according to the transtheoretical model of behavioral change [19]; and (3) calculate the most convenient time to send a motivational message to support smoking cessation for each patient. Specifically, the AI system used to tailor motivational messages is a hybrid HRS, whose design has been presented by Hors-Fraile et al [20].

The motivational messages were designed after conducting semistructured interviews involving two smoking cessation experts (a pulmonologist and a psychologist). Based on their comments, we identified the following five different topics, which were the most relevant for participants to succeed in their smoking cessation process: (1) general motivation; (2) healthy diet recommendations; (3) recommendations for an active lifestyle; (4) positive reinforcement messages to meet activity goals; and (5) benefits of being a nonsmoker. A total of 150 different messages were written for each topic by a health communication and health promotion PhD candidate, and they were validated by the two smoking cessation experts. This number of messages was chosen to ensure that, even in the worst case scenario where the AI system determines that a user needs to receive messages of a single topic, no user would get the same message twice during the intervention, as the maximum number of messages that could be received is 150 during the 1-year study. This approach minimizes any potential robotic and static feelings.

Messages included health communication and health promotion strategies, such as creating empathy, adding new knowledge, and changing existing misconceptions. The messages were short (less than 100 words), written in simple Spanish, written using a close and friendly tone, and written with easy-to-understand terms to facilitate comprehension by smokers of all educational levels. Other studies have used steps similar to the ones used by us for message creation [21]. However, in our design, the message content was not associated with any specific behavioral change model. Each message was checked to be suitable for all genders. When a message was clearly gender specific (ie, relation between smoking and erectile dysfunction in men), an alternate suitable message for the other gender was also designed (ie, risks of smoking during pregnancy).

HRSs involve self-learning algorithms that can adapt to the constantly evolving needs and interests of users and offer a high level of personalization of recommendations, taking advantage of the so-called “collective intelligence.” This is a new recommendation generation paradigm that contrasts the traditional tailoring approach, which provides static recommendations according to user responses to usually lengthy questionnaires. Thus, as this novel approach of tailoring is still in its infancy [22], it is of high interest to determine the relationship between the clinical outcomes of the So-Lo-Mo study and this type of technology.

The main objective of this So-Lo-Mo study was to compare usual psychopharmacological therapy (control group, CG) alone and alongside the aforementioned digital solution (intervention group, IG) for smoking cessation. The secondary objectives were to analyze health-related quality of life (HRQoL) and monitor healthy lifestyle and physical exercise habits. Complementarily, we assessed the impact of the AI-generated motivational messages on smoking cessation outcomes in the IG.

## Methods

### Study Design

This 12-month, randomized, open-label, parallel-group trial was performed at the Smoking Cessation Unit of Virgen del Rocío University Hospital in Seville (Spain) between October 24, 2016, and October 24, 2018, and it complied with the Declaration of Helsinki and Good Clinical Practice Guidelines. The recruitment period closed on October 23, 2017. The local ethics committee approved the study protocol, and written informed consent was obtained from each participant prior to inclusion. The clinical study design (NCT03553173) and technical study design (NCT03206619) have been published previously [23,24].

### Randomization

A technician generated a random-group table (n=240) using computer methods and following a 1:1 ratio between groups. Clinicians enrolled the participants and assigned them to the group mentioned in the table according to their enrolment sequence. Participants were blinded to this allocation, as those in the IG were told that the provided mobile app was part of usual care. Participants in the CG were not informed about the existence of the app and did not have access to it.

### Study Population

Smokers were recruited during routine visits to our outpatient clinic. The inclusion criteria were as follows: (1) age over 18 years and desire to stop smoking; (2) owning an Android smartphone (as the mobile app was only available for Android devices owing to time and resource constraints in the development phase of this study and Android phones were more likely to be owned by the target population owing to their lower entry price as compared with iPhones); and (3) ability to interact with the smartphone. Smartphone literacy was assessed by asking the participants if they commonly use other text exchange smartphone apps, such as Mail, SMS, and WhatsApp. The only exclusion criterion was any previous adverse effect related to the present pharmacological treatment.

### Power Calculation and Recruitment

A sample size of 236 was calculated during the study design phase, according to the following parameters: CI, 95%; statistical power, 80%; CG success rate, 35%; IG success rate, 55%; and expected dropout rate, 20%. A total of 240 participants were recruited and randomized for the stratified analysis.

### Intervention and Control Groups

**Figure 1:**
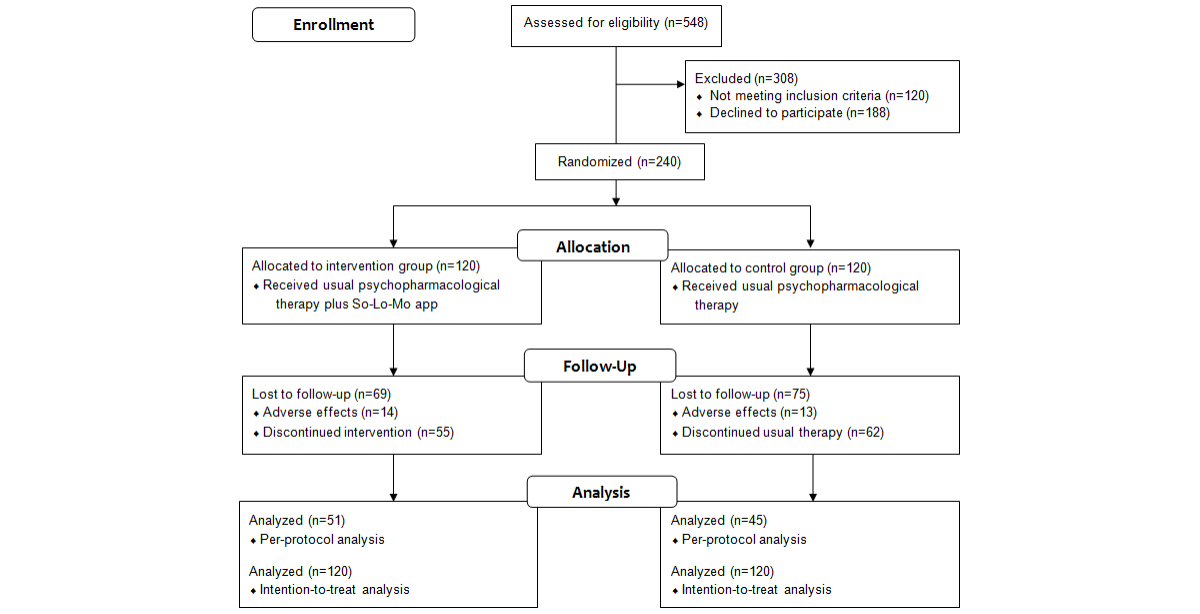
CONSORT diagram of the study.

**Figure 2:**
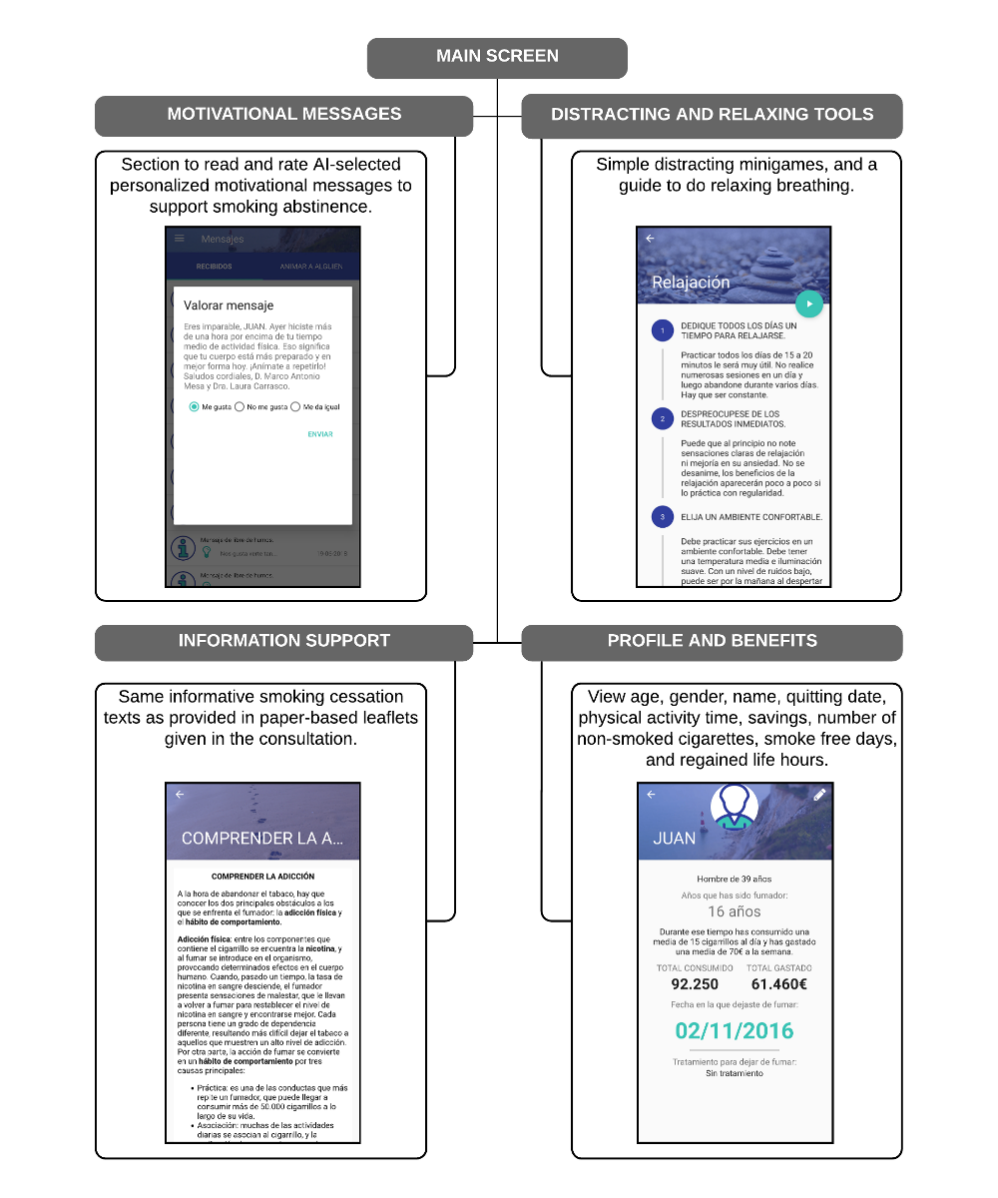
Structure of app features.

Usual care in the Smoking Cessation Unit of Virgen del Rocío University Hospital consists of pharmacological therapy with bupropion (ie, Zyntabac 150 mg; GlaxoSmithKline, Brentford, UK) or varenicline (ie, Champix 0.5 mg or 1 mg; Pfizer, New York, New York, USA) plus behavioral therapy. To facilitate recruitment and avoid bias associated with treatment cost, the SmokeFreeBrain project financed the drugs for usual care. Thus, all participants received their assigned treatments free of charge. Behavioral therapy, which was provided during face-to-face follow-up consultations, included various psychological techniques, including motivational interviews and cognitive-behavioral therapy. This psychopharmacological therapy was provided to both CG and IG participants. CG participants (n=120) received psychopharmacological therapy alone, whereas IG participants (n=120) received psychopharmacological therapy and used the digital therapeutic solution. Further information regarding enrollment, allocation, and the study analysis phases is provided in Figure 1. The app utilized behavioral techniques by sending personalized motivational messages generated using AI with the intent to achieve better smoking cessation rates by improving program adherence and abstinence rates [20]. After the participant read each message, the app asked the participant to rate how relevant the message was for him or her, collecting feedback for the AI. Complementing the core messaging feature, the mobile app had other minor sections. These included a user profile linked to the performed physical activity level collected by Google Fit (Google, Mountain View, California, USA), four smoking cessation benefit indicators (savings, smoke-free days, regained life hours by not smoking, and number of nonsmoked cigarettes since quitting), text-based information about smoking cessation, and a section containing relaxing and distracting elements (breathing exercises and minigames). Figure 2 presents an overview of the app features.

### Measurements

Information from all participants included demographic (age and sex) and socioeconomic data (profession and employment status), consumption history (daily cigarettes smoked, living with smokers, partner smoking status, smoking cessation attempts, etc), clinical information (weight, height, blood pressure, comorbidities, etc), nicotine dependence measured using the Fagerström test for nicotine dependence [25], and motivation to stop smoking according to the Richmond test [26]. Safety was measured as the number of adverse events related to pharmacological therapy.

The main clinical outcome was the 1-year smoking abstinence rate measured by exhaled carbon monoxide (CO), which was assessed using a CO tester (Micro+ Smokerlyzer; CoVita, Santa Barbara, California, USA) and urine cotinine tests. Participants with an exhaled CO level greater than 6 ppm were considered smokers [27]. Urine cotinine (SmokeScreen test; Concept Smoke Screen Ltd, Lincolnshire, UK) is a colorimetric test that measures the main nicotine metabolites, including cotinine. Participants with a cotinine concentration greater than 200 ng/ml were considered smokers [28]. Participants were considered smokers when at least one of the aforementioned conditions was met.

The HRQoL was assessed using the 36-item Short-Form Health Survey (SF-36) that was validated in Spanish [29] and the EuroQol 5-dimension 5-level (EuroQoL-5D-5L) questionnaire [30]. Physical activity was measured using the International Physical Activity Questionnaire (IPAQ) [31], and a healthy lifestyle was interpreted via body mass index (BMI) variations during follow-up consultations. A case report form, built upon the OpenClinica [32] tool, was developed to facilitate information management in the study. Information subsets were registered according to the following schedule: basal and 15, 30, 60, 90, 120, 180, and 365 (±5) days after the basal consultation [23].

The HRS impact on smoking cessation was assessed according to the precision of the recommendations sent by the system. Precision was calculated for both the message content recommendations and the time to open the messages, as the HRS aimed to optimize both variables. Further, we considered the generated engagement of the user with the system and the user appreciation of the messages as part of the technical evaluation of HRS impact. The starting point to measure the evolution of these metrics was the first day that a participant rated a message (December 5, 2016), and the assessment continued for the following 18 months to evaluate the progress. Detailed descriptions of these metric calculations are presented below.

#### Precision of the System

This measurement focused on the system’s effectiveness in recommending relevant messages. The mobile app required the user to rate all read messages at least once; otherwise, the user could no longer interact with the app. There were three message rating options (like, dislike, and neutral). The ratings were then coded as 1 for “like,” −1 for “dislike,” and 0 for “neutral.” The precision metric was calculated by dividing the number of hits (messages rated as “like”) by the total number of sent messages (precision_p). A modification of this measurement was performed by considering “likes” and “neutrals” as hits (precision_p_n,). We used two variants of precision to obtain different perspectives on how the system performed in selecting relevant messages. The second approach focused on minimizing the negatively rated messages. Regardless of the variant, when we code the hits as 1 and misses as 0, this metric is in fact an arithmetic mean (we sum the number of hits and divide them by the total number of sent messages). The value of this metric ranges from 0 to 1. A higher value is associated with more system precision. This is because on sending messages at random, we expect 33% positive feedback, 33% neutral feedback, and 33% negative feedback. However, when the system learns and becomes more “intelligent,” the percentage of negative feedback will decrease over time, increasing the value resulting from this metric.

#### Time to Open Motivational Messages

This pertained to the arithmetic mean of the elapsed time between the message being sent and opened in a 30-day period. We assumed that this metric would decrease over time because the system was expected to learn and become more “intelligent” in predicting the best time for the user to open and read the sent message.

#### Engagement With the System

To measure system engagement, we included a time stamp in each sent message and compared it with the time stamp sent by the app to the server when the user opened the given message. Thus, the engagement metric was determined by the ratio of rated messages calculated as the total of all rated messages from one user divided by the total number of messages sent to that user. As we coded each message rated with a “1” value and divided the finding by the total number of sent messages, this ratio coincides with the arithmetic mean. Thus, we assume that a higher value is associated with more user interest in the message and, consequently, higher user engagement. As this metric measured the engagement of the user with the system and not the engagement of the system with the user, we considered the set date to stop smoking for each user as the point to start counting each month. After this calculation, we offset the start date for each user to the first day the user rated the first message. This allowed us to compare the average engagement of each user across time. Therefore, the results are presented according the relative months to the user set date to stop smoking (eg, M3 results are the results of the third month after a participant stopped smoking). However, for one participant, M3 could be April if the participant stopped smoking in January, but for another participant, it could be December if the participant stopped smoking in September. The measurement of this follow-up was performed for the 12-month period that each participant in the IG was enrolled in the study, as participants in the CG did not use the mobile app.

#### Subjective Quality of the System

System quality was determined by the answers in an anonymized five-level Likert-type appreciation questionnaire [24], making it impossible to link the answers to the individual participants. The questionnaire was completed by each participant at the end of the 1-year follow-up period. The questions concerned the HRS according to the Recommender Systems Questionnaire of User Experience (ResQue) model [33] and the content of the messages according to the I-Change model [34]. Of the 15 constructs of the ResQue model, 12 were represented in the questions, as the remaining three did not apply to our HRS (ie, purchase intention). All the answers reflected the level of agreement of the participant with the proposed topics (1 indicating “totally disagree” and 5 indicating “totally agree”). We assessed how participants perceived the quality of the system after 12 months of usage by observing their questionnaire response distributions, means, and SDs. Group comparisons were not feasible owing to the complete anonymity of the questionnaire. The aim was to measure the degree of possible improvement of the system in the following areas included in the questionnaire: quality of recommended items, interaction adequacy, interface adequacy, perceived ease of use, perceived usefulness, control/transparency, attitudes, behavioral intentions, and message content to influence smoking risk perception, confidence, social support, and coping action support.

### Statistical Analysis

Descriptive analyses of participant characteristics according to absolute and relative frequencies for qualitative variables and mean (SD) for quantitative variables were conducted. For the primary endpoint and secondary outcomes (BMI, physical activity, and HRQoL), analyses were conducted on a per-protocol basis. These analyses included all participants who adhered to the schedule of eight consultations (basal through 365 days after the basal consultation).

Regarding incomplete data analyses, homoscedasticity tests were conducted among groups of cases with identical missing data patterns to evaluate whether data were missing completely at random [35]. A multinomial regression analysis was performed to assess the variables influencing the probability of a participant dropping out as compared with the probability of the treatment being effective. A “no efficacy” category was set as the reference category. As multinomial model effects are relative to the reference category, to assess whether the explicative variable effects were different between the “dropout” and “efficacy” categories, the same model was adjusted using the “dropout” category as a reference. Consequently, whether the variable effects were different between the “dropout” and “efficacy” categories was assessed. The relative risk ratio (RRR) with 95% CI was used for each model.

Lost data analysis and multinomial logistic models showed different patterns in participants who dropped out as compared with the patterns in those who completed the study, regardless of treatment efficacy. Therefore, smoking abstinence at 1 year was analyzed using logistic regression models on a per-protocol basis and intention-to-treat basis, and the effect measures were the OR and 95% CI.

For the selection of logistic regression and multinomial logistic models in per-protocol (n=94) analysis and intention-to-treat (n=240) analysis, a two-stage strategy was adopted. In the first step, the variable importance was quantified using Random Forest [36] with the mean decrease in accuracy as the score. Variables with a score above 0.5 were included in an Akaike information criterion–based stepwise selection strategy. The following two restrictions were applied to the final models selected: (1) absence of a pattern in model residuals and (2) variables with a generalized variance inflation factor [37] above 5 were not allowed in order to avoid collinearity.

To determine whether the HRS metrics had an impact on the clinical outcomes, we divided IG participants in cessation and no cessation subgroups at 12 months of the intervention. Thereafter, we conducted *t* tests for the 12-month results of precision, time to rate messages, and engagement, analyzing each of these two subgroups.

## Results

### Characteristics

Both groups had similar baseline characteristics, except for the maximum abstinence time and smoking cessation attempts (Table 1).

**Table 1:** Participant baseline characteristics.

Characteristic		Intervention group (n=120)	Control group (n=120)	*P* value
Female, n (%)		65 (54.2)	52 (43.3)	.09
Age (years), mean (SD)		48.38 (9.49)	50.93 (10.85)	.05
Age at smoking onset (years), mean (SD)		16.94 (4.07)	16.67 (3.60)	.54
Daily cigarettes, mean (SD)		21.45 (8.97)	20.75 (9.39)	.44
Lives with smokers, n (%)		45 (37.5)	49 (40.8)	.60
Partner smokes, n (%)		71 (59.2)	65 (54.2)	.43
Smoking cessation attempts, mean (SD)		0.88 (1.08)	1.14 (1.07)	.045
Maximum abstinence time, mean (SD)		10.45 (25.49)	13.68 (25.08)	.003
Body mass index, mean (SD)		27.02 (4.91)	27.03 (6.46)	.73
Unemployed, n (%)		33 (27.5)	38 (31.7)	.48
**Previous treatments, n (%)**		46 (38.3)	44 (36.7)	.79
	Varenicline	13 (10.8)	15 (12.5)	.69
	Bupropion	14 (11.7)	16 (13.3)	.70
	Nicotine	13 (10.8)	15 (12.5)	.84
	Others	16 (13.3)	11 (9.2)	.31
Comorbidities, n (%)		46 (38.3)	59 (49.2)	.12
Charlson index, mean (SD)		0.83 (1.25)	1.08 (1.34)	.06
Fagerström score, mean (SD)		5.89 (1.91)	5.60 (1.99)	.38
Richmond, mean (SD)		9.32 (0.80)	9.28 (0.88)	.88

### Efficacy (Unadjusted): 1-Year Smoking Abstinence Rate

**Figure 3:**
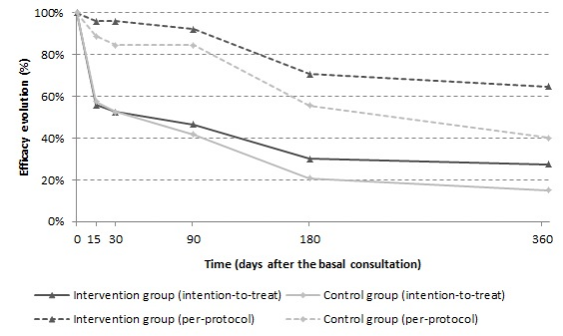
Efficacy evolution during the 1-year follow-up.

IG and CG participants who completed the study (per-protocol analysis) achieved efficacy rates of 64.7% (CI 51.6%-77.8%) and 40.0% (CI 25.7%-54.3%), respectively (*P*=.02; OR 2.75, CI 1.20-6.29) and a number needed to treat of 4 (CI 2.0-19.0). In the intention-to-treat analysis, IG and CG participants achieved efficacy rates of 27.5% (CI 19.5%-35.5%) and 15.0% (CI 8.6%-21.4%), respectively (*P*=.02; OR 2.15, CI 1.13-4.08) and a number needed to treat of 8 (CI 4.0-43.0). Figure 3 shows the efficacy evolution during the 1-year follow-up in each study group according to both analytic approaches.

### Body Mass Index and Physical Activity

BMI variances were similar for both groups. In the IG and CG, the mean BMI variances were 1.01 (CI 045-1.57) and 1.10 (CI 0.72-1.48), respectively, at 6 months and were 1.47 (CI 0.90-2.03) and 1.22 (CI 0.67-1.75), respectively, at 12 months. The between-group mean BMI differences at 6 and 12 months were −0.09 (CI −0.77 to 0.60; *P*=.80) and 0.25 (CI −0.53 to 1.03; *P*=.52), respectively.

According to the observed IPAQ scores, physical activity evolution patterns were similar in both study groups. Moreover, in the IG, 12.8% (6/47) and 25.5% (13/51) of participants increased their physical activity at 6 and 12 months, respectively, whereas 14.9% (7/47) and 13.7% (7/51) of participants reduced it at the corresponding points. On the other hand, in the CG, 14.6% (6/41) and 24.4% (11/45) of participants increased their physical activity at 6 and 12 months, respectively, whereas 24.4% (10/41) and 8.9% (4/45) of participants reduced it at the corresponding points. Table 2 summarizes the BMI and IPAQ results.

**Table 2:** Body mass index, physical activity and health-related quality of life variances in each group.

Variable		Intervention group^a^ (n=51)	Control group^a^ (n=45)	*P* value
**BMI^b^** **changes (kg/m^2^)**				
	At 6 months^c^	1.01 (0.45 to 1.57)	1.10 (0.72 to 1.48)	.80
	At 12 months	1.47 (0.90 to 2.03)	1.22 (0.67 to 1.75)	.52
**IPAQ^d^ changes at 6 months^c^**				
	Improvement	6 (12.8)	6 (14.6)	.47
	No change	34 (72.3)	25 (61.0)	
	Worsening	7 (14.9)	10 (24.4)	
**IPAQ changes at 12 months**				
	Improvement	13 (25.5)	11 (24.4)	.73
	No change	31 (60.8)	30 (66.7)	
	Worsening	7 (13.7)	4 (8.9)	
**VAS^e^ changes**				
	At 6 months^c^	4.04 (−0.76 to 8.84)	3.88 (−0.57 to 8.32)	.96
	At 12 months	5.78 (1.60 to 9.97)	2.78 (−1.86 to 7.41)	.33
**SF-36^f^ changes at 12 months**				
	Physical function	5.39 (2.18 to 8.61)	5.33 (1.43 to 9.24)	.98
	Physical role	2.82 (−0.47 to 6.10)	5.69 (0.43 to 10.96)	.34
	Body pain	3.78 (−1.52 to 9.09)	5.71 (−1.07 to 12.49)	.65
	General health	6.35 (1.83 to 10.87)	6.38 (0.73 to 12.02)	.99
	Vital	7.23 (1.92 to 12.54)	6.94 (1.75 to 12.14)	.94
	Social	0.74 (−4.86 to 6.33)	7.78 (2.82 to 12.73)	.06
	Emotional	4.90 (−0.22 to 10.02)	3.70 (−0.78 to 8.19)	.73
	Mental	3.33 (−2.12 to 8.79)	4.78 (−1.06 to 10.61)	.72

^a^Data are expressed as mean (95% CI) or n (%).

^b^BMI: body mass index.

^c^Data are missing.

^d^IPAQ: International Physical Activity Questionnaire.

^e^VAS: visual analog scale.

^f^SF-36: 36-item Short-Form Health Survey.

### Health-Related Quality of Life

Based on the EuroQoL-5D-5L questionnaire results, both groups had improved HRQoL scores at 6 months. Although the IG showed a greater improvement at 12 months, the difference was not statistically significant (Table 2). The mean between-group visual analog scale differences at 6 and 12 months were 0.17 (95% CI −6.36 to 6.70) and 3.01 (95% CI −3.14 to 9.16), respectively. Regarding the SF-36 dimensions, both groups showed improvement at 12 months, although there were no significant between-group differences. Table 2 summarizes the HRQoL results.

### Safety: Adverse Events

Nineteen adverse events (11/19 [58%] associated with bupropion and 8/19 [42%] with varenicline) were identified in 14 IG participants (11.7%), whereas 23 adverse events (10/23 [43%] associated with bupropion and 13/23 [57%] with varenicline) were identified in 13 CG participants (10.8%) (*P*=.84).

The most frequent events associated with bupropion were headache (6/21, 28.6%), insomnia (4/21, 19.0%), vertigo (4/21, 19.0%), acute abdominal pain (2/21, 9.5%), and others (5/21, 23.8%), and those associated with varenicline were nausea (6/21, 28.6%), acute abdominal pain (4/21, 19.0%), insomnia (3/21, 14.3%), vomiting (2/21, 9.5%), and others (6/21, 28.6%).

### Lost Data Pattern Analysis

The pattern of missing efficacy variable values was not totally random (*P*<.001). Multimedia Appendix 1 shows the variables whose distributions differed depending on the group; the values were not equally distributed between the participants who dropped out and those who completed the study. Table 3 shows the parameters estimated by the multinomial logistic regression model for each of the variable effectiveness levels (no efficacy, efficacy, and dropout) with respect to the reference category.

**Table 3:** Multinomial logistic regression.

Variable		RRR^a^	95% CI	*P* value
**Efficacy vs no efficacy**				
	Intercept	0.03	0.00-19.57	
	Body mass index	0.96	0.88-1.06	.42
	Maximum abstinence time	1.14	0.97-1.35	.11
	Fagerström score	0.99	0.74-1.31	.93
	Age at smoking onset	1.05	0.93-1.19	.43
	Daily cigarettes	0.99	0.93-1.06	.82
	Charlson index	1.00	0.72-1.39	.99
	Low physical activity	0.63	0.18-2.18	.47
	Medium physical activity	0.32	0.09-1.11	.07
	Richmond score	1.43	0.82-2.49	.21
	Group (intervention)	3.25	1.33-7.95	.01
	Drug (varenicline)	0.81	0.33-1.96	.64
**Dropout vs no efficacy**				
	Intercept	0.17	0.001-28.00	
	Body mass index	1.01	0.93-1.09	.88
	Maximum abstinence time	0.81	0.68-0.97	.03
	Fagerström score	1.31	1.02-1.68	.03
	Age at smoking onset	1.07	0.96-1.19	.23
	Daily cigarettes	1.00	0.96-1.06	.87
	Charlson index	0.86	0.65-1.13	.27
	Low physical activity	0.78	0.29-2.09	.62
	Medium physical activity	0.48	0.17-1.32	.15
	Richmond score	1.13	0.73-1.75	.59
	Group (intervention)	0.93	0.43-2.03	.85
	Drug (varenicline)	0.36	0.17-0.77	.01
**Efficacy vs dropout**				
	Intercept	0.18	0.001-61.91	
	Body mass index	0.96	0.88-1.04	.29
	Maximum abstinence time	1.41	1.18-1.67	<.001
	Fagerström score	0.75	0.58-0.97	.03
	Age at smoking onset	0.98	0.89-1.08	.73
	Daily cigarettes	0.99	0.94-1.05	.69
	Charlson index	1.17	0.87-1.57	.31
	Low physical activity	0.81	0.27-2.43	.71
	Medium physical activity	0.67	0.19-2.34	.53
	Richmond score	1.27	0.77-2.09	.36
	Group (intervention)	3.50	1.56-7.83	.002
	Drug (varenicline)	2.26	1.05-4.86	.04

^a^RRR: relative risk ratio.

Importantly, the probability of being in the “efficacy” category was higher for IG participants as compared with the “no efficacy” category (RRR=3.25; *P*=.01). Furthermore, increasing the baseline Fagerström score (higher nicotine dependence) in a unit rendered a smoker more likely to belong to the “dropout” category as compared with the “no efficacy” category (RRR=1.31; *P*=.03). Increasing the maximum smoking abstinence time reduced the probability of a smoker being in the “dropout” category as compared with the “no efficacy” category (RRR=0.81; *P*=.03). Furthermore, the probability of being in the “dropout” category increased for participants using bupropion as compared with the “no efficacy” category (RRR=0.36; *P*=.01).

### Efficacy (Adjusted): 1-Year Smoking Abstinence Rate

Table 4 shows the logistic regression analysis results for participants who completed the study (per-protocol basis). Notably, for the IG participants, the efficacy was 3.45 times higher with adjustment for age, motivation to stop smoking (Richmond scale), and comorbidity level. The efficacy probability decreased by 28.9% when a participant was not in the IG, with adjustment for the rest of the variables.

The results of the lost data pattern analysis and those obtained from the multinomial logistic models showed different patterns in participants who dropped out as compared with those who completed the study, regardless of treatment efficacy. However, as the variables associated with the dropout probability indicated a lack of treatment efficacy (belonging to the CG, short withdrawal period duration, fewer attempts, greater number of daily cigarettes, and baseline Fagerström score), a value of 0 (no efficacy) was assigned to participants who dropped out of the study. Therefore, the worst possible scenario models efficacy by intention-to-treat using a logistic regression model (Table 5).

These findings demonstrate that intervention efficacy was 3.13 higher with adjustment for the rest of the variables. Hence, the probability of long-term smoking cessation using the digital therapeutic solution was over three times higher than the probability of cessation with usual care alone.

**Table 4:** Logistic regression analysis of the 1-year smoking abstinence rate (per-protocol analysis).

Variable	OR	95% CI	*P* value
Intercept	0.00	0.00-0.02	—
Intervention group	3.45	1.39-9.13	.01
Age	1.09	1.03-1.17	.01
Motivation to stop smoking (Richmond score)	1.90	1.07-3.52	.03
Charlson index, medium comorbidity	0.76	0.16-3.60	.72
Charlson index, high comorbidity	0.07	0.01-0.45	.01

**Table 5:** Logistic regression analysis of the 1-year smoking abstinence rate (intention-to-treat analysis).

Variable	OR	95% CI	*P* value
Intercept	0	0.00-0.07	—
Group (intervention)	3.13	1.53-6.71	.002
Age	1.04	1.00-1.08	.04
Drug (varenicline)	1.49	0.74-3.04	.27
Maximum abstinence time	1.22	1.07-1.39	.004
Nicotine dependence (Fagerström score)	0.82	0.68-0.98	.03
Motivation for smoking cessation (Richmond score)	1.62	1.02-2.69	.049
Low physical activity (IPAQ^a^)	0.93	0.30-2.55	.89
Medium physical activity (IPAQ)	0.66	0.19-1.95	.47

^a^IPAQ: International Physical Activity Questionnaire.

### Health Recommender System Impact on Smoking Cessation Outcomes

A total of 17,111 messages and 2617 ratings were provided in the study, and there were 2311 ratings during the 12 months of follow up, 261 in the presmoking cessation phase, and 45 at 13 months or later, which were not part of this analysis.

#### Precision

**Figure 4:**
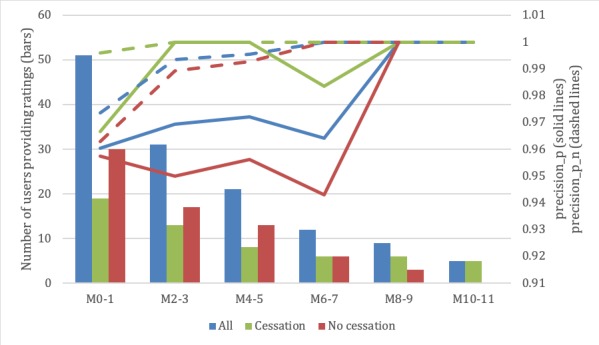
Evolution of health recommender system precision and number of users providing ratings by subgroup. M: month; precision_p: precision calculated by dividing the number of hits (messages rated as “like”) by the total number of sent messages; precision_p_n: precision calculated by dividing the number of hits (messages rated as “like” and “neutral”) by the total number of sent messages.

The *t* test results for each month indicated that there were no significant differences for system precision_p or precision_p_n in the cessation and no cessation subgroups. The detailed results are presented in Multimedia Appendix 2. A bimonthly graphical representation of the evolution of precision_p and precision_p_n over time is shown in Figure 4.

#### Time to Open Messages

**Figure 5:**
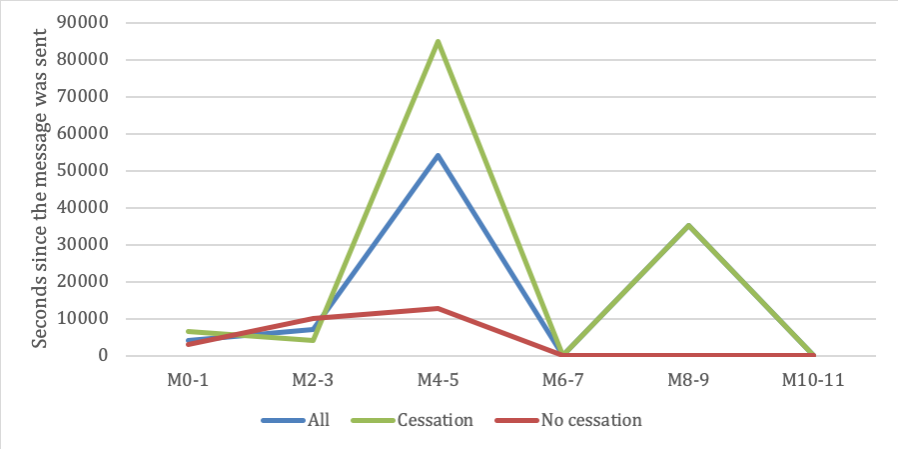
Mean time between a message being sent and opened. M: month.

We performed *t* tests (results are shown in Multimedia Appendix 2) and found no significant difference between the cessation and no cessation subgroups at any time point. A bimonthly graphical representation of the evolution of the time-to-open metric is shown in Figure 5.

#### Engagement

**Figure 6:**
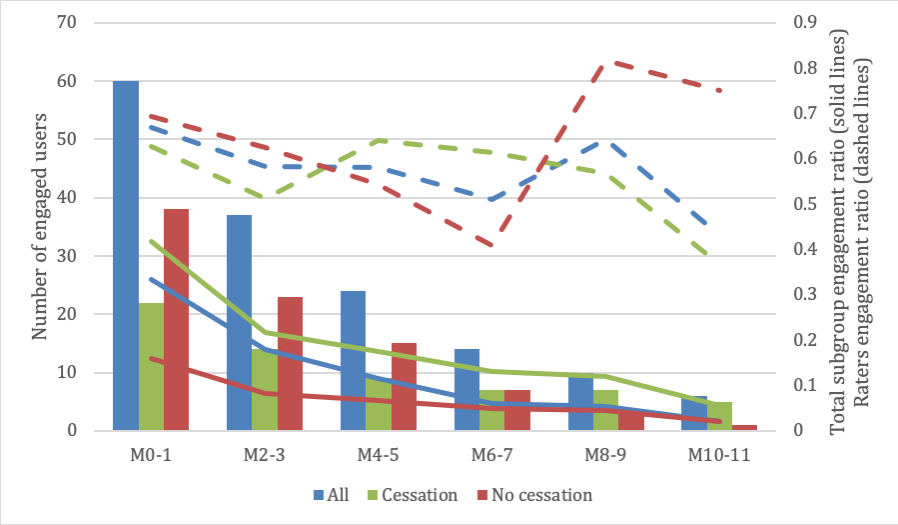
Evolution of user engagement. M: month.

The *t* tests shown in Multimedia Appendix 2 indicate that there were significant differences between the cessation and no cessation subgroups in only the first 7 (*P*=.04), 8 (*P*=.03), and 10 (*P*=.04) months, showing a trend toward higher engagement in the cessation subgroup. A bimonthly graphical representation of the evolution of the IG participants’ engagement with the system over time is shown in Figure 6.

#### Perceived Quality

Only 32 participants volunteered to respond to the questionnaire. However, not all participants who responded to the questionnaire completed it. The results are presented in Table 6.

**Table 6:** Answers to the perceived quality questionnaire.

Question	N	Minimum score	Maximum score	Mean	SD
The messages recommended to me matched my interests.	32	4	5	4.19	0.40
The messages recommended to me were novel.	32	3	5	4.06	0.50
The messages recommended to me were diverse.	32	4	5	4.47	0.51
The layout of the message interface was adequate	32	3	5	4.03	0.60
I found it easy to tell the system what I like/dislike.	32	4	5	4.34	0.48
I felt in control of modifying my interest profile.	32	3	5	3.63	0.66
I became familiar with the messaging system very quickly.	30	3	5	4.00	0.37
I understood why the messages were recommended to me.	31	3	5	4.03	0.48
The messages gave me good suggestions.	31	4	5	4.23	0.43
Overall, I am satisfied with the messages.	31	4	5	4.35	0.49
The messages can be trusted.	31	3	5	3.97	0.55
I would recommend the use of the message recommendations to my friends who smoke.	31	3	5	3.61	0.72
The messages convinced me that I am at risk for health problems if I do not quit smoking.	31	3	5	3.71	0.64
The messages convinced me that my smoking is a risky habit.	31	3	5	4.06	0.57
The messages convinced me of the advantages of smoking cessation.	30	3	5	3.67	0.66
The messages showed me how to get social support from others during cessation.	31	3	5	3.68	0.54
The messages helped me feel confident that I could successfully quit smoking.	31	3	5	4.00	0.26
The messages helped prepare actions to cope with difficult situations.	31	4	5	4.19	0.40

## Discussion

### Principal Findings

#### Clinical Outcomes

The combination of our digital therapeutic solution with psychopharmacological treatment was more effective in achieving tobacco abstinence at 12 months as compared with psychopharmacological treatment alone. According to the per-protocol analysis findings, abstinence was 2.75 times higher among IG participants than among CG participants (adjusted OR of 3.45 for age, motivation, and comorbidity level). According to the intention-to-treat analysis, abstinence was 2.15 times higher among IG participants than among CG participants (adjusted OR of 3.13 for age, drug used, maximum abstinence time, nicotine dependence, motivation, and physical activity). Moreover, our findings suggest that the probability of dropout increased for participants who used bupropion and had high nicotine dependence, whereas a high maximum smoking abstinence time reduced this probability.

Despite mobile apps offering several advantages over traditional smoking cessation methods, few studies have examined the content quality or the effectiveness of apps promoting smoking cessation [38-40]. To the best of our knowledge, this study provides the first scientific evidence of the efficacy of a behavioral change intervention via a mobile app powered by AI for smoking cessation at a 1-year follow-up. Furthermore, no other study has provided statistically significant results for a mobile intervention used in combination with smoking cessation drugs. Previous studies published their cessation results with 6 months of follow-up at the most, as described in the comparison with prior work subsection below. We provide evidence for the long-term impact of our digital therapeutic solution, with a follow-up period that is double of the follow-up period in previous studies. Further, this study is the first to analyze physical activity via the IPAQ after smoking cessation. Physical activity evolution patterns were similar in the IG and CG, with an increase in the percentage of participants showing improved physical activity and no significant difference between the IG and CG participants.

Weight gain during the first year of abstinence concerns smokers [41], and it may be a potential barrier to pursue smoking cessation. In this study, weight gain, measured by an increase in BMI, occurred in both groups. The app did not affect weight gain as there was no difference between the IG and CG.

A previous review showed that a higher number of cigarettes smoked was associated with a lower QoL. Additionally, a low QoL and depression were related with lower odds of successfully stopping smoking [42]. Participants who received treatments, including varenicline and bupropion, reported QoL improvements and increased abstinence duration as compared with those who did not receive pharmacotherapy [42,43]. The present results are comparable to these findings. There was a positive trend in the HRQoL scores reported by the IG, but with no relevant difference as compared with the scores in the CG.

#### Technical Outcomes

The complementary technical results showed that the HRS is able to learn the participants’ interests regarding the support message topics for smoking cessation. The system was more precise at the end of the intervention than at the beginning, as was expected for a recommender system. However, the minimum precision reached was very high (over 96%). We believe that there may be different reasons for this extremely high value. For instance, it could be due to the implemented hybrid HRS algorithm that mitigates the cold start problem and accurately recommends messages from the beginning according to a weighting formula described in a previous publication [20]. Additionally, it could be due to participants believing that their ratings would be viewed by health care professionals; hence, they modified their ratings toward a higher value than they otherwise would. This potential Hawthorne effect would be a limitation for digital health not identified in a recent study [44], as HRSs are only starting to be used in health care. Another reason may be that the system did not allow sufficient granularity for the users to vote; the participants were only afforded three rating options. A wider spread of rating options may have contributed to the differentiation of message relevance to a greater extent (for instance, separating fair, good, and entirely accurate messages). The statistical analysis did not find a significant difference in the precision achieved between participants who were successful in smoking cessation and those who were not successful. Hence, the achieved precision over time cannot be considered a predictor of smoking cessation, as the HRS recommended motivational messages equally well for all participants.

The time-to-open metric results showed that the HRS was not able to predict the best time to send a message for a decrease in the time between the message being sent and the user opening the message. This may be due to several factors influencing participant behavior other than the time they receive a message, which were not considered in the HRS. To improve the time, variables, such as the position of the phone, location, last activity, and cessation day, should be considered for inclusion as parameters in the HRS.

The generated engagement by the system showed statistical significance (*P*=.04) between the cessation and no cessation subgroups after the first 6 months of the intervention, favoring a higher engagement for those who managed to stop smoking. This is in line with the intuitive assumption that participants who engaged with the system to a greater extent received greater benefit and consequently were less likely to relapse. However, these differences were not significant at 9, 11, and 12 months owing to a decrease in the number of participants to such an extent at these points that the statistical power was limited.

As not all participants in the IG completed the perceived quality questionnaire and the questionnaire was anonymous, it is impossible to ensure that any conclusion derived from the collected results is not biased. However, the results showed a clearly positive perception of the quality of the system. Reasonably, only those who managed to stop smoking and presumably benefited from the system provided answers to the questionnaire, as they would have been more motivated to respond. On the other hand, participants who dropped out, probably due to relapse, would have not been interested in completing the questionnaire. However, it could also be argued that those who did not manage to stop smoking would have been willing to provide negative feedback on the system in the anonymized questionnaire. Nevertheless, the positive trend of the answers favors the first scenario. For similar research practices in the future, we suggest following a design that forces participants to respond to the questionnaire but still preserves their anonymity. This could be achieved by including the questionnaire in a previous stage of the trial to avoid an effect by the high dropout rate associated with digital interventions for smoking cessation.

The features of the presented AI-based digital therapeutic solution enabled the system to effectively support smoking cessation by providing support and advice for facilitating abstinence, enhance motivation, and clearly show a benefit [45]. The HRS was well perceived by the participants. Over time, it identified the most relevant motivational messages to send to each participant, and those who were engaged with the system to a great extent managed to successfully stop smoking by the end of the intervention. Therefore, this digital therapeutic solution may alleviate the known drawbacks of intensive complementary behavioral interventions for smoking cessation, which, despite their benefits, require extensive availability of well-trained professionals, leading to limited scalability, poor accessibility, and smoker resignation owing to long waiting times [46].

### Limitations

In previous similar studies, the dropout rate was usually very high, reflecting the difficulty of smoking cessation and high relapse rate [3]. In this study, the dropout rates were 57.5% and 62.5% in the IG and CG, respectively. A high dropout rate may bias the results; however, the dropout rate was similar between the groups, minimizing the possibility of bias. The efficacy of the intervention was verified by per-protocol and intention-to-treat analyses, providing a more realistic picture while reducing the importance of dropout or full treatment compliance. Moreover, an analysis of the characteristics of the dropout and no dropout categories within each group was carry out, and the same pattern was obtained between both groups (Multimedia Appendix 3). These findings suggest the potential benefit of the intervention, as participants did not experience abnormal adverse effects and were more likely to be abstinent after 1 year. Nevertheless, further exploration of its efficacy is needed for validation in other cultures, and studies should include a larger sample size and real-world data.

We found some random inconsistent values in the entries of the registered time, including duplicate entries and negative time values. The presented results correspond to filtered data (removing such values). Removal of these values reduced data quality for the metric, and the findings may not accurately represent the entire participant group. These inconsistent values may have been registered owing to glitches in communication between the smartphone app and the tracking system on the server.

Metrics for engagement at the aggregated level could not be provided as anticipated in the technical protocol because the software service used to track user data is not able to retrieve information from data older than 2 years. This information was not listed in the features of the service when it was selected for the purposes of the study. We encourage future researchers to use in-house user data tracking services to avoid relying on third-party software.

### Comparison With Prior Work

Previous studies have explored the use of mobile apps supporting smoking cessation. The SmartQuit study tested the feasibility, acceptability, preliminary efficacy, and mechanism of behavioral change of an innovative smartphone-delivered acceptance and commitment therapy app for smoking cessation in 196 adult participants and reported a successful cessation rate of 13% after 2 months of follow-up [47].

In another study, the authors assessed the efficacy of an interactive smoking cessation decision–aid app in 684 adults and reported a successful cessation rate of 10.2% [48]. Additionally, an app-based mindfulness training program for smoking cessation was assessed in 143 participants, and a successful cessation rate of 11.1% was achieved [49]. A parallel, double-blind, randomized, controlled, two-arm trial compared the efficacy of an evidence-informed smartphone app for smoking cessation (Crush the Crave) to that of an evidence-informed self-help guide (On the Road to Quitting) in 1599 subjects and reported successful cessation rates of 7.8% and 9.2%, respectively [50]. In all these studies, the abstinence rate was reported at 6 months from baseline. However, they did not include pharmacological treatment as part of the intervention, which is a clear difference from the approach in our study. It is remarkable that the dropout rates for both the IG and CG identified in this study are consistent with those previously reported in the literature [50].

Research has been performed on intervention efficacy for the use of an app specifically designed to support the smoking cessation process when added to pharmacological therapy. The preliminary efficacy of an app designed to prompt smokers to engage in physical activity was assessed at 6 months of follow-up in 44 regular smokers who received the app in addition to behavioral smoking cessation counselling, physical activity promotion, and pharmacological support, and the overall abstinence rates were 36% in an intention-to-treat analysis and 53% in a complete-case analysis [51]. In this previous study, the allocation of pharmacological treatment (varenicline and nicotine replacement therapy) was not controlled by the study protocol, which hinders the comparability of the results with those of our study. Additionally, in a randomized pilot study, a 12-week course of varenicline was prescribed to both arms to assess the feasibility and acceptability of the My Mobile Advice Program (MyMAP) smoking cessation app and estimate its effects on smoking cessation and medication adherence [52]; however, its efficacy could not be evidenced owing to the small sample size (n=33).

As stated in the conclusion section of a recent systematic review [53] that included 26 studies (n=33,849) involving text messaging and app-based smoking cessation interventions, there is moderate certainty evidence that automated text message–based smoking cessation interventions result in greater smoking cessation rates as compared with minimal smoking cessation support and there is moderate certainty evidence of the benefit of text messaging interventions in addition to other types of smoking cessation support as compared with smoking cessation support alone. The evidence of the comparison of smartphone apps with less intensive support had very low certainty, and further randomized controlled trials are needed to test these interventions. In this sense, the results of this study aim to contribute to an increase in the certainty level of the available evidence in this domain.

When focusing on the assessment of recommender systems, such as the one used in this study, the scientific community has extensively concentrated on improving the performance of recommender system algorithms with different metrics [54-58], mainly in nonhealth contexts, such as leisure [59] and e-commerce [60,61]. Among these approaches, common assessments for prediction accuracy are as follows: (1) mean absolute error and root mean squared error, as well as their normalized and averaged variants to predict ratings that users would give to items when the actual recommendation ratings of the items are known for the whole test set; (2) precision, recall, and false positive rate [58,62] for prediction of the usage of the recommendations; and (3) normalized discounted cumulative gain when the system presents a large list of elements (similar to a search in Google), where we expect that the most relevant elements are shown at the top of the list; and (4) coverage of the recommendation set [63]. Other authors have proposed the use of surveys to assess recommender systems, such as ResQue [33].

Regarding the evaluation of mobile Health (mHealth) apps for behavioral change, such as the one included in this study, some authors have proposed evaluation procedures, such as the National Institute for Health and Care Excellence adaptation [64], the Mobile App Rating Scale framework [65], and the Application Usage Factor, which is defined as the logarithm of the product of the number of active users of a mobile app and the median number of daily uses of the app [66]. Despite these attempts to set a methodological framework to assess mHealth apps, they still lack standardization and comprehensiveness [67]. In a recent study, McKay et al identified that there was no available related best practice [68]. Instead, they proposed the following generic guidelines for what these types of evaluations should include: (1) assessment of the quality of health-related content; (2) review of the usability and functionality of the app; and (3) critique of the app potential with regard to behavioral change promotion.

Despite the lack of consensus on specific evaluation methods, it is well accepted that engagement is a key element for mHealth solutions to be successful [69]. Engagement has been considered a positive early indicator for behavioral change [70], as the process of behavioral change requires time. Previous studies showed that technologies supporting reaching a specific behavior health goal help people stick to the desired goal [71,72]. Further, Scherer et al [73] found a significant positive correlation between engagement and fewer dropouts in an mHealth intervention. A recent approach to provide an engagement score for a mHealth app is the Engagement Index proposed by Taki et al [74]. To calculate the score, the authors combined the number of pages in the app that a participant visited each day, number of app accesses during the program, number of push notifications opened, elapsed time between app accesses, and subjective answers to a questionnaire. However, achieving user engagement is difficult [75], and its level could be improved [76-78]. Therefore, it seems reasonable to consider engagement as a key metric for digital health solutions. However, the definition of engagement varies among studies. For instance, Iacoviello et al [79] considered the number of times users opened an app, number of interactions they had, and number of weeks in which users had at least one interaction with the program as indicators of engagement. Owen et al [80] determined engagement by calculating several variables, such as the number of downloads and number of sessions (a session being basically opening of the app). Yet, it is common to just use opening the app as an indicator of engagement, as adopted in previous studies [40,81]. All these engagement interpretations fall under the categorization of “system usage data” proposed by Short et al [82], which is most frequently used. However, other engagement measurement approaches have been proposed, such as ecological momentary assessments, psychophysiological measurements, and qualitive methods.

In our So-Lo-Mo study, the HRS was intuitively related to user engagement, as some studies have shown that good and timely recommendations to stop smoking motivate users to read more future recommendations [74,83]. Hence, we proposed a combination of qualitative and quantitative metrics, which measure the intended purpose of the system (quality of the recommendations, engagement of the participants with the system, and participants’ perception of the system). To our knowledge, this is the first assessment involving a HRS. However, in the case of smoking cessation, low engagement may not necessarily result in low impact on behavioral change, as some studies have previously shown the “gateway effect” [84] and “happy abandonment” [85]. There are several reasons for this, including the burden of the required interactions as compared with perceived outcomes or the internalization of health habits denoting that support is perceived as no longer necessary [86]. Consequently, we needed to carefully analyze the engagement of users across time points keeping in mind all these factors to extract any conclusions. Thus, our system’s intended usage [82] is expected to be higher during the first weeks of the smoking cessation process and to progressively decrease until there is no engagement with the system, which will be reflected in engagement measurements.

### Conclusions

The So-Lo-Mo intervention offers a promising strategy for smoking cessation. The use of this digital therapeutic solution alongside pharmacological treatment was much more efficacious in achieving tobacco abstinence as compared with pharmacological treatment alone at the 1-year follow-up. However, this intervention did not improve participant HRQoL and physical activity levels.

Analysis of the impact of the HRS showed that participants benefited from the recommendations, and those who engaged with the system were more likely to succeed in smoking cessation. Therefore, the proposed HRS had a positive impact on the participants and offered them personalized and relevant messages, although it could not determine when supportive messages should be sent to minimize the time until they are read.

Health care providers should consider incorporating this digital therapeutic solution in their usual care, as it can facilitate positive outcomes for participants willing to stop smoking.

## Appendix

Multimedia Appendix 1 Incomplete data analysis.

Multimedia Appendix 2 Health recommender system performance analysis, Precision_p.

Multimedia Appendix 3 Characteristics of the dropout and non-dropout within each group.

Multimedia Appendix 4 CONSORT-EHEALTH checklist (V 1.6.1).

## Abbreviations

AI: artificial intelligence
BMI: body mass index
CG: control group
EuroQoL-5D-5L: EuroQol 5-dimension 5-level
HRS: health recommender system
IG: intervention group
IPAQ: International Physical Activity Questionnaire
ResQue: Recommender Systems Questionnaire of User Experience
RRR: relative risk ratio
SF-36: 36-item Short Form
