# Supplementary material for: A Mobile Health Solution Complementing Psychopharmacology-Supported Smoking Cessation: Randomized Controlled Trial
Source: JMIR Mhealth Uhealth. 2020 Apr 27;8(4):e17530. doi: 10.2196/17530 (PMC7215523; doi:10.2196/17530)
Supplement: Multimedia Appendix 1 [file mhealth_v8i4e17530_app1.doc]

Appendix 1. Incomplete data analysis

|  | Non-dropout (n=96) | Dropout (n=144) | P value |
| --- | --- | --- | --- |
| Last session |  |  | < 0.001 |
| Mean (CI) | 8.00 (8.00, 8.00) | 3.09 (2.79, 3.39) |  |
| Median (Q1, Q3) | 8.00 (8.00, 8.00) | 3.00 (2.00, 4.00) |  |
| Age |  |  | 0.730 |
| Mean (CI) | 50.06 (48.17, 51.95) | 49.38 (47.60, 51.17) |  |
| Median (Q1, Q3) | 50.50 (43.00, 56.00) | 49.00 (43.00, 57.00) |  |
| Age at start |  |  | 0.855 |
| Mean (CI) | 16.74 (15.96, 17.52) | 16.85 (16.21, 17.48) |  |
| Median (Q1, Q3) | 16.00 (14.00, 18.00) | 16.00 (14.00, 18.00) |  |
| Daily cigarettes |  |  | 0.004 |
| Mean (CI) | 19.20 (17.44, 20.96) | 22.37 (20.84, 23.90) |  |
| Median (Q1, Q3) | 20.00 (11.50, 20.00) | 20.00 (20.00, 30.00) |  |
| Quitting attemps |  |  | < 0.001 |
| Mean (CI) | 1.26 (1.02, 1.50) | 0.84 (0.68, 1.00) |  |
| Median (Q1, Q3) | 1.00 (1.00, 1.00) | 1.00 (0.00, 1.00) |  |
| Maximum abstinence time |  |  | < 0.001 |
| Mean (CI) | 17.96 (11.46, 24.46) | 8.10 (5.03, 11.16) |  |
| Median (Q1, Q3) | 6.00 (1.00, 12.75) | 1.00 (0.00, 10.50) |  |
| Body mass index |  |  | 0.788 |
| Mean (CI) | 26.77 (25.90, 27.64) | 27.19 (26.12, 28.27) |  |
| Median (Q1, Q3) | 26.27 (24.42, 28.83) | 25.86 (23.51, 29.83) |  |
| Charlson index |  |  | 0.540 |
| Mean (CI) | 1.02 (0.75, 1.30) | 0.91 (0.70, 1.12) |  |
| Median (Q1, Q3) | 1.00 (0.00, 1.00) | 1.00 (0.00, 1.00) |  |
| Richmond test |  |  | 0.104 |
| Mean (CI) | 9.40 (9.23, 9.56) | 9.23 (9.09, 9.37) |  |
| Median (Q1, Q3) | 10.00 (9.00, 10.00) | 9.00 (9.00, 10.00) |  |
| Fagerström test |  |  | < 0.001 |
| Mean (CI) | 5.15 (4.75, 5.54) | 6.15 (5.84, 6.45) |  |
| Median (Q1, Q3) | 5.00 (4.00, 6.00) | 6.00 (5.00, 8.00) |  |
| Adverse events |  |  | < 0.001 |
| No; n (%) | 96 (100.0%) | 117 (81.2%) |  |
| Yes; n (%) | 0 (0.0%) | 27 (18.8%) |  |
| Group |  |  | 0.429 |
| Control; n (%) | 45 (46.9%) | 75 (52.1%) |  |
| Intervention; n (%) | 51 (53.1%) | 69 (47.9%) |  |
| Gender |  |  | 0.958 |
| Female; n (%) | 47 (49.0%) | 70 (48.6%) |  |
| Male; n (%) | 49 (51.0%) | 74 (51.4%) |  |
| Live in smokers |  |  | 0.517 |
| No; n (%) | 56 (58.3%) | 90 (62.5%) |  |
| Yes; n (%) | 40 (41.7%) | 54 (37.5%) |  |
| Partner smokers |  |  | 0.523 |
| No; n (%) | 44 (45.8%) | 60 (41.7%) |  |
| Yes; n (%) | 52 (54.2%) | 84 (58.3%) |  |
| Unemployed |  |  | 0.908 |
| No; n (%) | 68 (70.8%) | 101 (70.1%) |  |
| Yes; n (%) | 28 (29.2%) | 43 (29.9%) |  |
| Previous Vareniclina |  |  | 0.250 |
| No; n (%) | 82 (85.4%) | 130 (90.3%) |  |
| Yes; n (%) | 14 (14.6%) | 14 (9.7%) |  |
| Previous Bupropion |  |  | 0.232 |
| No; n (%) | 87 (90.6%) | 123 (85.4%) |  |
| Yes; n (%) | 9 (9.4%) | 21 (14.6%) |  |

Appendix 1. Incomplete data analysis (continued)

|  | Non-dropout (n=96) | Dropout (n=144) | P value |
| --- | --- | --- | --- |
| Previous nicotine |  |  | 0.417 |
| No; n (%) | 87 (90.6%) | 125 (86.8%) |  |
| Yes; n (%) | 9 (9.4%) | 19 (13.2%) |  |
| Previous others |  |  | 0.934 |
| No; n (%) | 85 (88.5%) | 128 (88.9%) |  |
| Yes; n (%) | 11 (11.5%) | 16 (11.1%) |  |
| Drug |  |  | 0.008 |
| Bupropion; n (%) | 38 (39.6%) | 82 (56.9%) |  |
| Varenicline; n (%) | 58 (60.4%) | 62 (43.1%) |  |
| Charlson (comorbidity level) |  |  | 0.624 |
| Without comorbidity; n (%) | 73 (76.0%) | 115 (79.9%) |  |
| Medium comorbidity; n (%) | 14 (14.6%) | 15 (10.4%) |  |
| High comorbidity; n (%) | 9 (9.4%) | 14 (9.7%) |  |
| Physical activity |  |  | 0.227 |
| Low; n (%) | 15 (15.6%) | 29 (20.1%) |  |
| Medium; n (%) | 16 (16.7%) | 14 (9.7%) |  |
| High; n (%) | 65 (67.7%) | 101 (70.1%) |  |
