# Supplementary material for: A Mobile Health Solution Complementing Psychopharmacology-Supported Smoking Cessation: Randomized Controlled Trial
Source: JMIR Mhealth Uhealth. 2020 Apr 27;8(4):e17530. doi: 10.2196/17530 (PMC7215523; doi:10.2196/17530)
Supplement: Multimedia Appendix 2 [file mhealth_v8i4e17530_app2.docx]

# Appendix 2: Health Recommender System performance analysis

## Precision_p

| **Group Statistics** | | | | | |
| --- | --- | --- | --- | --- | --- |
|  |  | N | Mean | Std. Deviation | Std. Error Mean |
| precision_p_relative_m1 | No Cessation | 36 | ,969502 | ,0701522 | ,0116920 |
|  | Cessation | 16 | ,930556 | ,2496912 | ,0624228 |
| precision_p_relative_m2 | No Cessation | 30 | ,945705 | ,1904725 | ,0347754 |
|  | Cessation | 16 | ,977679 | ,0892857 | ,0223214 |
| precision_p_relative_m3 | No Cessation | 20 | ,952368 | ,1390560 | ,0310939 |
|  | Cessation | 12 | 1,000000 | ,0000000 | ,0000000 |
| precision_p_relative_m4 | No Cessation | 17 | ,952941 | ,0975810 | ,0236669 |
|  | Cessation | 12 | 1,000000 | ,0000000 | ,0000000 |
| precision_p_relative_m5 | No Cessation | 13 | ,973626 | ,0654254 | ,0181457 |
|  | Cessation | 8 | 1,000000 | ,0000000 | ,0000000 |
| precision_p_relative_m6 | No Cessation | 10 | ,933333 | ,1405457 | ,0444444 |
|  | Cessation | 7 | 1,000000 | ,0000000 | ,0000000 |
| precision_p_relative_m7 | No Cessation | 6 | ,904762 | ,2332847 | ,0952381 |
|  | Cessation | 7 | 1,000000 | ,0000000 | ,0000000 |
| precision_p_relative_m8 | No Cessation | 4 | 1,000000 | ,0000000 | ,0000000 |
|  | Cessation | 6 | ,969697 | ,0742270 | ,0303030 |
| precision_p_relative_m9 | No Cessation | 3 | 1,000000 | ,0000000^a^ | ,0000000 |
|  | Cessation | 6 | 1,000000 | ,0000000^a^ | ,0000000 |
| precision_p_relative_m10 | No Cessation | 2 | 1,000000 | ,0000000^a^ | ,0000000 |
|  | Cessation | 4 | 1,000000 | ,0000000^a^ | ,0000000 |
| precision_p_relative_m11 | No Cessation | 1 | 1,000000 | . | . |
|  | Cessation | 3 | 1,000000 | ,0000000 | ,0000000 |
| precision_p_relative_m12 | No Cessation | 1 | 1,000000 | . | . |
|  | Cessation | 4 | 1,000000 | ,0000000 | ,0000000 |
| a. t cannot be computed because the standard deviations of both groups are 0. | | | | | |

| **Independent Samples Test** | | | | | | | | | | |
| --- | --- | --- | --- | --- | --- | --- | --- | --- | --- | --- |
|  | | Levene's Test for Equality of Variances | | t-test for Equality of Means | | | | | | |
|  |  | F | Sig. | t | df | Sig. (2-tailed) | Mean Difference | Std. Error Difference | 95% Confidence Interval of the Difference | |
|  |  |  |  |  |  |  |  |  | Lower | Upper |
| precision_p_relative_m1 | Equal variances assumed | 3,848 | ,055 | ,871 | 50 | ,388 | ,0389466 | ,0447161 | -,0508683 | ,1287615 |
|  | Equal variances not assumed |  |  | ,613 | 16,062 | ,548 | ,0389466 | ,0635083 | -,0956425 | ,1735357 |
| precision_p_relative_m2 | Equal variances assumed | 1,428 | ,238 | -,633 | 44 | ,530 | -,0319736 | ,0505171 | -,1337842 | ,0698370 |
|  | Equal variances not assumed |  |  | -,774 | 43,532 | ,443 | -,0319736 | ,0413228 | -,1152795 | ,0513322 |
| precision_p_relative_m3 | Equal variances assumed | 6,244 | ,018 | -1,179 | 30 | ,248 | -,0476316 | ,0404087 | -,1301572 | ,0348941 |
|  | Equal variances not assumed |  |  | -1,532 | 19,000 | ,142 | -,0476316 | ,0310939 | -,1127118 | ,0174486 |
| precision_p_relative_m4 | Equal variances assumed | 15,303 | ,001 | -1,662 | 27 | ,108 | -,0470588 | ,0283222 | -,1051712 | ,0110536 |
|  | Equal variances not assumed |  |  | -1,988 | 16,000 | ,064 | -,0470588 | ,0236669 | -,0972304 | ,0031127 |
| precision_p_relative_m5 | Equal variances assumed | 7,360 | ,014 | -1,129 | 19 | ,273 | -,0263736 | ,0233643 | -,0752757 | ,0225285 |
|  | Equal variances not assumed |  |  | -1,453 | 12,000 | ,172 | -,0263736 | ,0181457 | -,0659098 | ,0131625 |
| precision_p_relative_m6 | Equal variances assumed | 10,980 | ,005 | -1,243 | 15 | ,233 | -,0666667 | ,0536499 | -,1810186 | ,0476853 |
|  | Equal variances not assumed |  |  | -1,500 | 9,000 | ,168 | -,0666667 | ,0444444 | -,1672070 | ,0338737 |
| precision_p_relative_m7 | Equal variances assumed | 7,404 | ,020 | -1,088 | 11 | ,300 | -,0952381 | ,0875028 | -,2878305 | ,0973543 |
|  | Equal variances not assumed |  |  | -1,000 | 5,000 | ,363 | -,0952381 | ,0952381 | -,3400554 | ,1495792 |
| precision_p_relative_m8 | Equal variances assumed | 4,000 | ,081 | ,800 | 8 | ,447 | ,0303030 | ,0378788 | -,0570456 | ,1176517 |
|  | Equal variances not assumed |  |  | 1,000 | 5,000 | ,363 | ,0303030 | ,0303030 | -,0475934 | ,1081995 |
| precision_p_relative_m11 | Equal variances assumed | . | . | . | 2 | . | ,0000000 | ,0000000 | ,0000000 | ,0000000 |
|  | Equal variances not assumed |  |  | . | . | . | ,0000000 | . | . | . |
| precision_p_relative_m12 | Equal variances assumed | . | . | . | 3 | . | ,0000000 | ,0000000 | ,0000000 | ,0000000 |
|  | Equal variances not assumed |  |  | . | . | . | ,0000000 | . | . | . |

## Precision_p_n

| **Group Statistics** | | | | | |
| --- | --- | --- | --- | --- | --- |
|  |  | N | Mean | Std. Deviation | Std. Error Mean |
| precision_p_n_relative_m1 | No Cessation | 36 | ,974443 | ,0657415 | ,0109569 |
|  | Cessation | 16 | ,985243 | ,0404033 | ,0101008 |
| precision_p_n_relative_m2 | No Cessation | 30 | ,951587 | ,1892906 | ,0345596 |
|  | Cessation | 16 | ,995536 | ,0178571 | ,0044643 |
| precision_p_n_relative_m3 | No Cessation | 20 | 1,000000 | ,0000000^a^ | ,0000000 |
|  | Cessation | 12 | 1,000000 | ,0000000^a^ | ,0000000 |
| precision_p_n_relative_m4 | No Cessation | 17 | ,977941 | ,0660743 | ,0160254 |
|  | Cessation | 12 | 1,000000 | ,0000000 | ,0000000 |
| precision_p_n_relative_m5 | No Cessation | 13 | 1,000000 | ,0000000^a^ | ,0000000 |
|  | Cessation | 8 | 1,000000 | ,0000000^a^ | ,0000000 |
| precision_p_n_relative_m6 | No Cessation | 10 | ,983333 | ,0527046 | ,0166667 |
|  | Cessation | 7 | 1,000000 | ,0000000 | ,0000000 |
| precision_p_n_relative_m7 | No Cessation | 6 | 1,000000 | ,0000000^a^ | ,0000000 |
|  | Cessation | 7 | 1,000000 | ,0000000^a^ | ,0000000 |
| precision_p_n_relative_m8 | No Cessation | 4 | 1,000000 | ,0000000^a^ | ,0000000 |
|  | Cessation | 6 | 1,000000 | ,0000000^a^ | ,0000000 |
| precision_p_n_relative_m9 | No Cessation | 3 | 1,000000 | ,0000000^a^ | ,0000000 |
|  | Cessation | 6 | 1,000000 | ,0000000^a^ | ,0000000 |
| precision_p_n_relative_m10 | No Cessation | 2 | 1,000000 | ,0000000^a^ | ,0000000 |
|  | Cessation | 4 | 1,000000 | ,0000000^a^ | ,0000000 |
| precision_p_n_relative_m11 | No Cessation | 1 | 1,000000 | . | . |
|  | Cessation | 3 | 1,000000 | ,0000000 | ,0000000 |
| precision_p_n_relative_m12 | No Cessation | 1 | 1,000000 | . | . |
|  | Cessation | 4 | 1,000000 | ,0000000 | ,0000000 |
| a. t cannot be computed because the standard deviations of both groups are 0. | | | | | |

| **Independent Samples Test** | | | | | | | | | | |
| --- | --- | --- | --- | --- | --- | --- | --- | --- | --- | --- |
|  | | Levene's Test for Equality of Variances | | t-test for Equality of Means | | | | | | |
|  |  | F | Sig. | t | df | Sig. (2-tailed) | Mean Difference | Std. Error Difference | 95% Confidence Interval of the Difference | |
|  |  |  |  |  |  |  |  |  | Lower | Upper |
| precision_p_n_relative_m1 | Equal variances assumed | 1,254 | ,268 | -,606 | 50 | ,547 | -,0108002 | ,0178139 | -,0465804 | ,0249800 |
|  | Equal variances not assumed |  |  | -,725 | 44,603 | ,472 | -,0108002 | ,0149024 | -,0408225 | ,0192221 |
| precision_p_n_relative_m2 | Equal variances assumed | 3,496 | ,068 | -,922 | 44 | ,362 | -,0439484 | ,0476823 | -,1400458 | ,0521490 |
|  | Equal variances not assumed |  |  | -1,261 | 29,960 | ,217 | -,0439484 | ,0348467 | -,1151189 | ,0272221 |
| precision_p_n_relative_m4 | Equal variances assumed | 6,527 | ,017 | -1,150 | 27 | ,260 | -,0220588 | ,0191776 | -,0614080 | ,0172904 |
|  | Equal variances not assumed |  |  | -1,376 | 16,000 | ,188 | -,0220588 | ,0160254 | -,0560311 | ,0119134 |
| precision_p_n_relative_m6 | Equal variances assumed | 3,474 | ,082 | -,828 | 15 | ,420 | -,0166667 | ,0201187 | -,0595487 | ,0262153 |
|  | Equal variances not assumed |  |  | -1,000 | 9,000 | ,343 | -,0166667 | ,0166667 | -,0543693 | ,0210360 |
| precision_p_n_relative_m11 | Equal variances assumed | . | . | . | 2 | . | ,0000000 | ,0000000 | ,0000000 | ,0000000 |
|  | Equal variances not assumed |  |  | . | . | . | ,0000000 | . | . | . |
| precision_p_n_relative_m12 | Equal variances assumed | . | . | . | 3 | . | ,0000000 | ,0000000 | ,0000000 | ,0000000 |
|  | Equal variances not assumed |  |  | . | . | . | ,0000000 | . | . | . |

## Time to open

| **Group Statistics** | | | | | |
| --- | --- | --- | --- | --- | --- |
|  |  | N | Mean | Std. Deviation | Std. Error Mean |
| Mean_Time_To_Open_Relative_M1 | No Cessation | 33 | 1287,9522 | 1727,45193 | 300,71078 |
|  | Cessation | 9 | 1476,1062 | 834,96099 | 278,32033 |
| Mean_Time_To_Open_Relative_M2 | No Cessation | 26 | 3805,0214 | 9806,78396 | 1923,26857 |
|  | Cessation | 16 | 9199,9174 | 11319,36515 | 2829,84129 |
| Mean_Time_To_Open_Relative_M3 | No Cessation | 13 | 10004,4727 | 16394,84923 | 4547,11304 |
|  | Cessation | 13 | 4282,5681 | 3688,83719 | 1023,09936 |
| Mean_Time_To_Open_Relative_M4 | No Cessation | 1 | 1104,5556 | . | . |
|  | Cessation | 5 | 1914,0714 | 1754,24710 | 784,52315 |
| Mean_Time_To_Open_Relative_M5 | No Cessation | 2 | 11510,8750 | 13936,89788 | 9854,87500 |
|  | Cessation | 4 | 85307,9375 | 91209,71023 | 45604,85512 |
| Mean_Time_To_Open_Relative_M6 | No Cessation | 1 | 15517,6667 | . | . |
|  | Cessation | 1 | 1144,0000 | . | . |
| Mean_Time_To_Open_Relative_M7 | No Cessation | 0^a^ | . | . | . |
|  | Cessation | 0^a^ | . | . | . |
| Mean_Time_To_Open_Relative_M8 | No Cessation | 0^a^ | . | . | . |
|  | Cessation | 0^a^ | . | . | . |
| Mean_Time_To_Open_Relative_M9 | No Cessation | 0^a^ | . | . | . |
|  | Cessation | 0^a^ | . | . | . |
| Mean_Time_To_Open_Relative_M10 | No Cessation | 0^a^ | . | . | . |
|  | Cessation | 1 | 35349,8571 | . | . |
| Mean_Time_To_Open_Relative_M11 | No Cessation | 0^a^ | . | . | . |
|  | Cessation | 0^a^ | . | . | . |
| Mean_Time_To_Open_Relative_M12 | No Cessation | 0^a^ | . | . | . |
|  | Cessation | 0^a^ | . | . | . |
| a. t cannot be computed because at least one of the groups is empty. | | | | | |

| **Independent Samples Test** | | | | | | | | | | |
| --- | --- | --- | --- | --- | --- | --- | --- | --- | --- | --- |
|  | | Levene's Test for Equality of Variances | | t-test for Equality of Means | | | | | | |
|  |  | F | Sig. | t | df | Sig. (2-tailed) | Mean Difference | Std. Error Difference | 95% Confidence Interval of the Difference | |
|  |  |  |  |  |  |  |  |  | Lower | Upper |
| Mean_Time_To_Open_Relative_M1 | Equal variances assumed | ,130 | ,721 | -,315 | 40 | ,755 | -188,15397 | 597,75555 | -1396,26300 | 1019,95505 |
|  | Equal variances not assumed |  |  | -,459 | 28,030 | ,650 | -188,15397 | 409,74282 | -1027,43320 | 651,12525 |
| Mean_Time_To_Open_Relative_M2 | Equal variances assumed | 3,018 | ,090 | -1,633 | 40 | ,110 | -5394,89595 | 3304,48340 | -12073,50603 | 1283,71413 |
|  | Equal variances not assumed |  |  | -1,577 | 28,419 | ,126 | -5394,89595 | 3421,54405 | -12398,95336 | 1609,16145 |
| Mean_Time_To_Open_Relative_M3 | Equal variances assumed | 8,432 | ,008 | 1,228 | 24 | ,231 | 5721,90453 | 4660,79063 | -3897,49456 | 15341,30361 |
|  | Equal variances not assumed |  |  | 1,228 | 13,212 | ,241 | 5721,90453 | 4660,79063 | -4330,73049 | 15774,53954 |
| Mean_Time_To_Open_Relative_M3 | Equal variances assumed | . | . | -,421 | 4 | ,695 | -809,51587 | 1921,68141 | -6144,95882 | 4525,92708 |
|  | Equal variances not assumed |  |  | . | . | . | -809,51587 | . | . | . |
| Mean_Time_To_Open_Relative_M5 | Equal variances assumed | 185,533 | ,000 | -1,075 | 4 | ,343 | -73797,06250 | 68672,96253 | -264463,77320 | 116869,64820 |
|  | Equal variances not assumed |  |  | -1,582 | 3,265 | ,205 | -73797,06250 | 46657,48998 | -215683,73060 | 68089,60555 |
| Mean_Time_To_Open_Relative_M6 | Equal variances assumed | . | . | . | 0 | . | 14373,66667 | . | . | . |
|  | Equal variances not assumed |  |  | . | . | . | 14373,66667 | . | . | . |

## Engagement

| **Group Statistics** | | | | | |
| --- | --- | --- | --- | --- | --- |
|  |  | N | Mean | Std. Deviation | Std. Error Mean |
| engagement_individual_level_m1 | No Cessation | 69 | ,456965 | ,6580964 | ,0792255 |
|  | Cessation | 31 | ,364067 | ,4845447 | ,0870268 |
| engagement_individual_level_m2 | No Cessation | 69 | ,259833 | ,4463945 | ,0537396 |
|  | Cessation | 31 | ,332477 | ,4612055 | ,0828349 |
| engagement_individual_level_m3 | No Cessation | 69 | ,215098 | ,4177741 | ,0502941 |
|  | Cessation | 31 | ,235226 | ,4003170 | ,0718991 |
| engagement_individual_level_m4 | No Cessation | 69 | ,162191 | ,4005420 | ,0482196 |
|  | Cessation | 31 | ,194087 | ,3628439 | ,0651687 |
| engagement_individual_level_m5 | No Cessation | 70 | ,120539 | ,3240798 | ,0387350 |
|  | Cessation | 31 | ,172191 | ,3883684 | ,0697530 |
| engagement_individual_level_m6 | No Cessation | 70 | ,077642 | ,2198902 | ,0262819 |
|  | Cessation | 31 | ,190188 | ,4387221 | ,0787968 |
| engagement_individual_level_m7 | No Cessation | 69 | ,041010 | ,1504572 | ,0181129 |
|  | Cessation | 31 | ,135064 | ,3053773 | ,0548474 |
| engagement_individual_level_m8 | No Cessation | 69 | ,030100 | ,1584258 | ,0190722 |
|  | Cessation | 31 | ,133717 | ,3057441 | ,0549133 |
| engagement_individual_level_m9 | No Cessation | 69 | ,038780 | ,1984652 | ,0238924 |
|  | Cessation | 31 | ,110227 | ,2818438 | ,0506206 |
| engagement_individual_level_m10 | No Cessation | 69 | ,014755 | ,1135463 | ,0136694 |
|  | Cessation | 31 | ,110215 | ,3450458 | ,0619721 |
| engagement_individual_level_m11 | No Cessation | 68 | ,009804 | ,0808452 | ,0098039 |
|  | Cessation | 31 | ,063453 | ,2044608 | ,0367222 |
| engagement_individual_level_m12 | No Cessation | 68 | ,012255 | ,1010565 | ,0122549 |
|  | Cessation | 31 | ,035980 | ,1217240 | ,0218623 |

| **Independent Samples Test** | | | | | | | | | | |
| --- | --- | --- | --- | --- | --- | --- | --- | --- | --- | --- |
|  | | Levene's Test for Equality of Variances | | t-test for Equality of Means | | | | | | |
|  |  | F | Sig. | t | df | Sig. (2-tailed) | Mean Difference | Std. Error Difference | 95% Confidence Interval of the Difference | |
|  |  |  |  |  |  |  |  |  | Lower | Upper |
| engagement_individual_level_m1 | Equal variances assumed | 1,140 | ,288 | ,704 | 98 | ,483 | ,0928978 | ,1319442 | -,1689413 | ,3547368 |
|  | Equal variances not assumed |  |  | ,789 | 76,998 | ,432 | ,0928978 | ,1176875 | -,1414480 | ,3272435 |
| engagement_individual_level_m2 | Equal variances assumed | ,124 | ,726 | -,745 | 98 | ,458 | -,0726433 | ,0975106 | -,2661500 | ,1208634 |
|  | Equal variances not assumed |  |  | -,736 | 56,177 | ,465 | -,0726433 | ,0987399 | -,2704294 | ,1251428 |
| engagement_individual_level_m3 | Equal variances assumed | ,138 | ,711 | -,226 | 98 | ,822 | -,0201273 | ,0891923 | -,1971266 | ,1568720 |
|  | Equal variances not assumed |  |  | -,229 | 60,184 | ,819 | -,0201273 | ,0877438 | -,1956300 | ,1553753 |
| engagement_individual_level_m4 | Equal variances assumed | ,060 | ,806 | -,379 | 98 | ,706 | -,0318961 | ,0841936 | -,1989755 | ,1351833 |
|  | Equal variances not assumed |  |  | -,393 | 63,450 | ,695 | -,0318961 | ,0810684 | -,1938758 | ,1300837 |
| engagement_individual_level_m5 | Equal variances assumed | 1,614 | ,207 | -,694 | 99 | ,489 | -,0516525 | ,0743935 | -,1992653 | ,0959604 |
|  | Equal variances not assumed |  |  | -,647 | 49,316 | ,520 | -,0516525 | ,0797865 | -,2119634 | ,1086584 |
| engagement_individual_level_m6 | Equal variances assumed | 12,166 | ,001 | -1,720 | 99 | ,089 | -,1125461 | ,0654465 | -,2424060 | ,0173139 |
|  | Equal variances not assumed |  |  | -1,355 | 36,848 | ,184 | -,1125461 | ,0830643 | -,2808737 | ,0557816 |
| engagement_individual_level_m7 | Equal variances assumed | 14,752 | ,000 | -2,068 | 98 | ,041 | -,0940547 | ,0454858 | -,1843199 | -,0037895 |
|  | Equal variances not assumed |  |  | -1,628 | 36,708 | ,112 | -,0940547 | ,0577608 | -,2111207 | ,0230113 |
| engagement_individual_level_m8 | Equal variances assumed | 19,815 | ,000 | -2,234 | 98 | ,028 | -,1036170 | ,0463898 | -,1956761 | -,0115579 |
|  | Equal variances not assumed |  |  | -1,782 | 37,434 | ,083 | -,1036170 | ,0581310 | -,2213556 | ,0141216 |
| engagement_individual_level_m9 | Equal variances assumed | 6,077 | ,015 | -1,454 | 98 | ,149 | -,0714468 | ,0491384 | -,1689603 | ,0260667 |
|  | Equal variances not assumed |  |  | -1,276 | 43,894 | ,209 | -,0714468 | ,0559758 | -,1842664 | ,0413728 |
| engagement_individual_level_m10 | Equal variances assumed | 17,808 | ,000 | -2,072 | 98 | ,041 | -,0954600 | ,0460663 | -,1868772 | -,0040428 |
|  | Equal variances not assumed |  |  | -1,504 | 32,956 | ,142 | -,0954600 | ,0634617 | -,2245804 | ,0336604 |
| engagement_individual_level_m11 | Equal variances assumed | 14,897 | ,000 | -1,874 | 97 | ,064 | -,0536488 | ,0286221 | -,1104556 | ,0031581 |
|  | Equal variances not assumed |  |  | -1,411 | 34,351 | ,167 | -,0536488 | ,0380084 | -,1308621 | ,0235646 |
| engagement_individual_level_m12 | Equal variances assumed | 3,169 | ,078 | -1,015 | 97 | ,313 | -,0237252 | ,0233772 | -,0701225 | ,0226720 |
|  | Equal variances not assumed |  |  | -,947 | 49,621 | ,348 | -,0237252 | ,0250628 | -,0740748 | ,0266243 |
