# Supplementary material for: A Mobile Health Solution Complementing Psychopharmacology-Supported Smoking Cessation: Randomized Controlled Trial
Source: JMIR Mhealth Uhealth. 2020 Apr 27;8(4):e17530. doi: 10.2196/17530 (PMC7215523; doi:10.2196/17530)
Supplement: Multimedia Appendix 3 [file mhealth_v8i4e17530_app3.doc]

Appendix 3. Characteristics of the dropout and non-dropout within each group

|  | **Intervention group** | | | **Control group** | | |
| --- | --- | --- | --- | --- | --- | --- |
|  | **Non-dropout (N=51)** | **Dropout (N=69)** | **p value** | **Non-dropout (N=45)** | **Dropout (N=75)** | **p value** |
| Last session |  |  | < 0.001 |  |  | < 0.001 |
| Mean (CI) | 8.00 (8.00, 8.00) | 2.81 (2.38, 3.24) |  | 8.00 (8.00, 8.00) | 3.35 (2.92, 3.78) |  |
| Median (Q1, Q3) | 8.00 (8.00, 8.00) | 2.00 (2.00, 4.00) |  | 8.00 (8.00, 8.00) | 3.00 (2.00, 4.50) |  |
| Age |  |  | 0.529 |  |  | 0.955 |
| Mean (CI) | 49.12 (46.68, 51.56) | 47.84 (45.42, 50.26) |  | 51.13 (48.12, 54.15) | 50.80 (48.18, 53.42) |  |
| Median (Q1, Q3) | 49.00 (42.50, 55.00) | 48.00 (43.00, 54.00) |  | 52.00 (45.00, 57.00) | 51.00 (43.50, 58.00) |  |
| Age at start |  |  | 0.505 |  |  | 0.268 |
| Mean (CI) | 17.29 (16.04, 18.55) | 16.68 (15.78, 17.59) |  | 16.11 (15.24, 16.99) | 17.00 (16.09, 17.91) |  |
| Median (Q1, Q3) | 16.00 (15.00, 18.00) | 16.00 (14.00, 18.00) |  | 15.00 (14.00, 17.00) | 16.00 (14.00, 18.00) |  |
| Daily cigarettes |  |  | 0.002 |  |  | 0.296 |
| Mean (CI) | 18.75 (16.43, 21.06) | 23.45 (21.28, 25.62) |  | 19.71 (16.93, 22.49) | 21.37 (19.19, 23.55) |  |
| Median (Q1, Q3) | 20.00 (12.00, 20.00) | 20.00 (20.00, 30.00) |  | 20.00 (10.00, 20.00) | 20.00 (15.00, 22.50) |  |
| Quitting attemps |  |  | 0.007 |  |  | 0.027 |
| Mean (CI) | 1.16 (0.80, 1.51) | 0.67 (0.45, 0.88) |  | 1.38 (1.05, 1.71) | 1.00 (0.76, 1.24) |  |
| Median (Q1, Q3) | 1.00 (0.50, 1.00) | 0.00 (0.00, 1.00) |  | 1.00 (1.00, 2.00) | 1.00 (0.00, 1.00) |  |
| Maximum abstinence time |  |  | < 0.001 |  |  | 0.07 |
| Mean (CI) | 19.48 (9.31, 29.65) | 3.77 (1.77, 5.77) |  | 16.23 (8.11, 24.36) | 12.14 (6.61, 17.66) |  |
| Median (Q1, Q3) | 5.00 (0.00, 12.00) | 0.00 (0.00, 2.00) |  | 6.00 (2.00, 15.00) | 3.00 (0.00, 12.00) |  |
| Body mass index |  |  | 0.568 |  |  | 0.869 |
| Mean (CI) | 27.33 (25.97, 28.70) | 26.79 (25.59, 27.99) |  | 26.14 (25.07, 27.20) | 27.57 (25.80, 29.33) |  |
| Median (Q1, Q3) | 26.30 (24.53, 29.64) | 25.66 (23.46, 29.98) |  | 26.23 (24.35, 28.04) | 25.86 (23.58, 29.40) |  |
| Charlson index |  |  | 0.518 |  |  | 0.726 |
| Mean (CI) | 0.92 (0.54, 1.30) | 0.75 (0.47, 1.04) |  | 1.13 (0.72, 1.55) | 1.05 (0.75, 1.36) |  |
| Median (Q1, Q3) | 1.00 (0.00, 1.00) | 0.00 (0.00, 1.00) |  | 1.00 (0.00, 2.00) | 1.00 (0.00, 1.50) |  |
| Richmond test |  |  | 0.096 |  |  | 0.523 |
| Mean (CI) | 9.43 (9.20, 9.66) | 9.23 (9.04, 9.42) |  | 9.36 (9.11, 9.60) | 9.23 (9.01, 9.44) |  |
| Median (Q1, Q3) | 10.00 (9.00, 10.00) | 9.00 (9.00, 10.00) |  | 10.00 (9.00, 10.00) | 9.00 (9.00, 10.00) |  |
| Fagerström test |  |  | < 0.001 |  |  | 0.07 |
| Mean (CI) | 5.14 (4.63, 5.64) | 6.45 (6.02, 6.88) |  | 5.16 (4.51, 5.80) | 5.87 (5.44, 6.29) |  |
| Median (Q1, Q3) | 5.00 (4.00, 6.00) | 6.00 (5.00, 8.00) |  | 5.00 (4.00, 7.00) | 6.00 (4.50, 7.00) |  |
| Adverse events |  |  | < 0.001 |  |  | 0.003 |
| No; n (%) | 51 (100.0%) | 55 (79.7%) |  | 45 (100.0%) | 62 (82.7%) |  |
| Yes; n (%) | 0 (0.0%) | 14 (20.3%) |  | 0 (0.0%) | 13 (17.3%) |  |
| Gender |  |  | 0.105 |  |  | 0.087 |
| Female; n (%) | 32 (62.7%) | 33 (47.8%) |  | 15 (33.3%) | 37 (49.3%) |  |
| Male; n (%) | 19 (37.3%) | 36 (52.2%) |  | 30 (66.7%) | 38 (50.7%) |  |
| Live in smokers |  |  | 0.962 |  |  | 0.314 |
| No; n (%) | 32 (62.7%) | 43 (62.3%) |  | 24 (53.3%) | 47 (62.7%) |  |
| Yes; n (%) | 19 (37.3%) | 26 (37.7%) |  | 21 (46.7%) | 28 (37.3%) |  |
| Partner smokers |  |  | 0.414 |  |  | 0.887 |
| No; n (%) | 23 (45.1%) | 26 (37.7%) |  | 21 (46.7%) | 34 (45.3%) |  |
| Yes; n (%) | 28 (54.9%) | 43 (62.3%) |  | 24 (53.3%) | 41 (54.7%) |  |
| Unemployed |  |  | 0.402 |  |  | 0.478 |
| No; n (%) | 39 (76.5%) | 48 (69.6%) |  | 29 (64.4%) | 53 (70.7%) |  |
| Yes; n (%) | 12 (23.5%) | 21 (30.4%) |  | 16 (35.6%) | 22 (29.3%) |  |
| Drug |  |  | 0.016 |  |  | 0.187 |
| Bupropion | 19 (37.3%) | 41 (59.4%) |  | 19 (42.2%) | 41 (54.7%) |  |
| Varenicline | 32 (62.7%) | 28 (40.6%) |  | 26 (57.8%) | 34 (45.3%) |  |
| Previous Vareniclina |  |  | 0.381 |  |  | 0.433 |
| No; n (%) | 44 (86.3%) | 63 (91.3%) |  | 38 (84.4%) | 67 (89.3%) |  |
| Yes; n (%) | 7 (13.7%) | 6 (8.7%) |  | 7 (15.6%) | 8 (10.7%) |  |
| Previous Bupropion |  |  | 0.09 |  |  | 1 |
| No; n (%) | 48 (94.1%) | 58 (84.1%) |  | 39 (86.7%) | 65 (86.7%) |  |
| Yes; n (%) | 3 (5.9%) | 11 (15.9%) |  | 6 (13.3%) | 10 (13.3%) |  |
| Previous nicotine |  |  | 0.554 |  |  | 0.722 |
| No; n (%) | 47 (92.2%) | 60 (87.0%) |  | 40 (88.9%) | 65 (86.7%) |  |
| Yes; n (%) | 4 (7.8%) | 9 (13.0%) |  | 5 (11.1%) | 10 (13.3%) |  |

Appendix 3. Analysis of the dropout and non-dropout within each group (continued)

|  | **Intervention group** | | | **Control group** | | |
| --- | --- | --- | --- | --- | --- | --- |
|  | **Non-dropout (N=51)** | **Dropout (N=69)** | **p value** | **Non-dropout (N=45)** | **Dropout (N=75)** | **p value** |
| Previous others |  |  | 0.913 |  |  | 0.935 |
| No; n (%) | 44 (86.3%) | 60 (87.0%) |  | 41 (91.1%) | 68 (90.7%) |  |
| Yes; n (%) | 7 (13.7%) | 9 (13.0%) |  | 4 (8.9%) | 7 (9.3%) |  |
| Charlson (comorbidity level) |  |  | 0.684 |  |  | 0.803 |
| Without comorbidity; n (%) | 41 (80.4%) | 59 (85.5%) |  | 32 (71.1%) | 56 (74.7%) |  |
| Medium comorbidity; n (%) | 6 (11.8%) | 5 (7.2%) |  | 8 (17.8%) | 10 (13.3%) |  |
| High comorbidity; n (%) | 4 (7.8%) | 5 (7.2%) |  | 5 (11.1%) | 9 (12.0%) |  |
| IPAQScoreS1Cat_0 |  |  | 0.808 |  |  | 0.252 |
| Low | 7 (13.7%) | 11 (15.9%) |  | 8 (17.8%) | 18 (24.0%) |  |
| Medium | 7 (13.7%) | 7 (10.1%) |  | 9 (20.0%) | 7 (9.3%) |  |
| High | 37 (72.5%) | 51 (73.9%) |  | 28 (62.2%) | 50 (66.7%) |  |
