# Supplementary material for: A Mobile Health Solution Complementing Psychopharmacology-Supported Smoking Cessation: Randomized Controlled Trial
Source: JMIR Mhealth Uhealth. 2020 Apr 27;8(4):e17530. doi: 10.2196/17530 (PMC7215523; doi:10.2196/17530)
Supplement: Multimedia Appendix 4 [file mhealth_v8i4e17530_app4.pdf]

CONSORT-EHEALTH (V 1.6.1) - Submission/Publication Form

The CONSORT-EHEALTH checklist is intended for authors of randomized trials evaluating web-based and Internet-based applications/interventions, including mobile interventions, electronic games (incl multiplayer games), social media, certain telehealth applications, and other interactive and/or networked electronic applications. Some of the items (e.g. all subitems under item 5 - description of the intervention) may also be applicable for other study designs.

The goal of the CONSORT EHEALTH checklist and guideline is to be
a) a guide for reporting for authors of RCTs,
b) to form a basis for appraisal of an ehealth trial (in terms of validity)

CONSORT-EHEALTH items/subitems are MANDATORY reporting items for studies published in the Journal of Medical Internet Research and other journals / scientific societies endorsing the checklist.

Items numbered 1., 2., 3., 4a., 4b etc are original CONSORT or CONSORT-NPT (non-pharmacologic treatment) items.
Items with Roman numerals (i., ii, iii, iv etc.) are CONSORT-EHEALTH extensions/clarifications.

As the CONSORT-EHEALTH checklist is still considered in a formative stage, we would ask that you also RATE ON A SCALE OF 1-5 how important/useful you feel each item is FOR THE PURPOSE OF THE CHECKLIST and reporting guideline (optional).

Mandatory reporting items are marked with a red *.
In the textboxes, either copy & paste the relevant sections from your manuscript into this form - please include any quotes from your manuscript in QUOTATION MARKS,
or answer directly by providing additional information not in the manuscript, or elaborating on why the item was not relevant for this study.

YOUR ANSWERS WILL BE PUBLISHED AS A SUPPLEMENTARY FILE TO YOUR PUBLICATION IN JMIR AND ARE CONSIDERED PART OF YOUR PUBLICATION (IF ACCEPTED).
Please fill in these questions diligently. Information will not be copyedited, so please use proper spelling and grammar, use correct capitalization, and avoid abbreviations.

DO NOT FORGET TO SAVE AS PDF _AND_ CLICK THE SUBMIT BUTTON SO YOUR ANSWERS ARE IN OUR DATABASE !!!

Citation Suggestion (if you append the pdf as Appendix we suggest to cite this paper in the caption):
Eysenbach G, CONSORT-EHEALTH Group
CONSORT-EHEALTH: Improving and Standardizing Evaluation Reports of Web-based and [illegible]

Você está editando sua resposta. O compartilhamento deste URL permitirá que outras pessoas também a editem.

INSERIR UMA NOVA RESPOSTA

URL: [illegible]

doi: 10.2196/jmir.1923
PMID: 22209829

*Obrigatório

Your name *

First Last

Francisco José Núñez-Benjumea

Primary Affiliation (short), City, Country *

University of Toronto, Toronto, Canada

Salumedia Labs, Seville, Spain

Your e-mail address *

abc@gmail.com

kikonunez@salumedia.com

Title of your manuscript *

Provide the (draft) title of your manuscript.

Mobile Health Solution Complementing Psychopharmacology-Supported Smoking Cessation: Randomized Controlled Trial

Name of your App/Software/Intervention *

If there is a short and a long/alternate name, write the short name first and add the long name in brackets.

DigiQuit

Você está editando sua resposta. O compartilhamento deste URL permitirá que outras pessoas também a editem.

INSERIR UMA NOVA RESPOSTA

Evaluated Version (if any)

e.g. "V1", "Release 2017-03-01", "Version 2.0.27913"

Sua resposta

Language(s) *

What language is the intervention/app in? If multiple languages are available, separate by comma (e.g. "English, French")

Spanish

URL of your Intervention Website or App

e.g. a direct link to the mobile app on app in appstore (itunes, Google Play), or URL of the website. If the intervention is a DVD or hardware, you can also link to an Amazon page.

https://www.digiquit.com/

URL of an image/screenshot (optional)

Sua resposta

Accessibility *

Can an enduser access the intervention presently?

- ( ) access is free and open
- ( ) access only for special usergroups, not open
- ( ) access is open to everyone, but requires payment/subscription/in-app purchases
- (x) app/intervention no longer accessible
- ( ) Outro:

Você está editando sua resposta. O compartilhamento deste URL permitirá que outras pessoas também a editem. INSERIR UMA NOVA RESPOSTA

Primary Medical Indication/Disease/Condition *

e.g. "Stress", "Diabetes", or define the target group in brackets after the condition, e.g. "Autism (Parents of children with)", "Alzheimers (Informal Caregivers of)"

Smoking Cessation

Primary Outcomes measured in trial *

comma-separated list of primary outcomes reported in the trial

Efficacy, Efficiency

Secondary/other outcomes

Are there any other outcomes the intervention is expected to affect?

To monitor the safety of usual psychopharmacological therapies (varenicline, bupropion, behavioral therapy), To monitor healthy lifestyle and
physical exercise habits.

Recommended "Dose" *

What do the instructions for users say on how often the app should be used?

- ○ Approximately Daily
- ○ Approximately Weekly
- ○ Approximately Monthly
- ○ Approximately Yearly
- ◉ "as needed"
- ○ Outro:

Você está editando sua resposta. O compartilhamento deste URL permitirá que outras pessoas também a editem. INSERIR UMA NOVA RESPOSTA

Approx. Percentage of Users (starters) still using the app as recommended after 3 months *

- ( ) unknown / not evaluated
- ( ) 0-10%
- ( ) 11-20%
- ( ) 21-30%
- ( ) 31-40%
- ( ) 41-50%
- ( ) 51-60%
- (•) 61-70%
- ( ) 71%-80%
- ( ) 81-90%
- ( ) 91-100%
- ( ) Outro:

Overall, was the app/intervention effective? *

- (•) yes: all primary outcomes were significantly better in intervention group vs control
- ( ) partly: SOME primary outcomes were significantly better in intervention group vs control
- ( ) no statistically significant difference between control and intervention
- ( ) potentially harmful: control was significantly better than intervention in one or more outcomes
- ( ) inconclusive: more research is needed
- ( ) Outro:

Você está editando sua resposta. O compartilhamento deste URL permitirá que outras pessoas também a editem.

INSERIR UMA NOVA RESPOSTA

Article Preparation Status/Stage *

At which stage in your article preparation are you currently (at the time you fill in this form)

- ( ) not submitted yet - in early draft status
- ( ) not submitted yet - in late draft status, just before submission
- ( ) submitted to a journal but not reviewed yet
- ( ) submitted to a journal and after receiving initial reviewer comments
- (x) submitted to a journal and accepted, but not published yet
- ( ) published
- ( ) Outro:

Journal *

If you already know where you will submit this paper (or if it is already submitted), please provide the journal name (if it is not JMIR, provide the journal name under "other")

- ( ) not submitted yet / unclear where I will submit this
- ( ) Journal of Medical Internet Research (JMIR)
- (x) JMIR mHealth and UHealth
- ( ) JMIR Serious Games
- ( ) JMIR Mental Health
- ( ) JMIR Public Health
- ( ) JMIR Formative Research
- ( ) Other JMIR sister journal
- ( ) Outro:

Você está editando sua resposta. O compartilhamento deste URL permitirá que outras pessoas também a editem.

INSERIR UMA NOVA RESPOSTA

Is this a full powered effectiveness trial or a pilot/feasibility trial? *

- ○ Pilot/feasibility
- ◉ Fully powered

Manuscript tracking number *

If this is a JMIR submission, please provide the manuscript tracking number under "other" (The ms tracking number can be found in the submission acknowledgement email, or when you login as author in JMIR. If the paper is already published in JMIR, then the ms tracking number is the four-digit number at the end of the DOI, to be found at the bottom of each published article in JMIR)

- ○ no ms number (yet) / not (yet) submitted to / published in JMIR
- ◉ Outro: #17530

TITLE AND ABSTRACT

1a) TITLE: Identification as a randomized trial in the title

1a) Does your paper address CONSORT item 1a? *

I.e does the title contain the phrase "Randomized Controlled Trial"? (if not, explain the reason under "other")

- ◉ yes
- ○ Outro:

Você está editando sua resposta. O compartilhamento deste URL permitirá que outras pessoas também a editem.

INSERIR UMA NOVA RESPOSTA

1a-i) Identify the mode of delivery in the title

Identify the mode of delivery. Preferably use “web-based” and/or “mobile” and/or “electronic game” in the title. Avoid ambiguous terms like “online”, “virtual”, “interactive”. Use “Internet-based” only if Intervention includes non-web-based Internet components (e.g. email), use “computer-based” or “electronic” only if offline products are used. Use “virtual” only in the context of “virtual reality” (3-D worlds). Use “online” only in the context of “online support groups”. Complement or substitute product names with broader terms for the class of products (such as “mobile” or “smart phone” instead of “iphone”), especially if the application runs on different platforms.

	1	2	3	4	5	
subitem not at all important	○	○	○	○	◉	essential

Does your paper address subitem 1a-i? *

Copy and paste relevant sections from manuscript title (include quotes in quotation marks "like this" to indicate direct quotes from your manuscript), or elaborate on this item by providing additional information not in the ms, or briefly explain why the item is not applicable/relevant for your study

"Mobile Health Solution"

1a-ii) Non-web-based components or important co-interventions in title

Mention non-web-based components or important co-interventions in title, if any (e.g., “with telephone support”).

	1	2	3	4	5	
subitem not at all important	○	○	○	○	◉	essential

Does your paper address subitem 1a-ii?

Copy and paste relevant sections from manuscript title (include quotes in quotation marks "like this" to indicate direct quotes from your manuscript), or elaborate on this item by providing additional information not in the ms, or briefly explain why the item is not applicable/relevant for your study

"Complementing Psychopharmacology-Supported Smoking Cessation"

Você está editando sua resposta. O compartilhamento deste URL permitirá que outras pessoas também a editem.

INSERIR UMA NOVA RESPOSTA

1a-iii) Primary condition or target group in the title

Mention primary condition or target group in the title, if any (e.g., “for children with Type I Diabetes”) Example: A Web-based and Mobile Intervention with Telephone Support for Children with Type I Diabetes: Randomized Controlled Trial

	1	2	3	4	5	
subitem not at all important	○	○	○	○	◉	essential

Does your paper address subitem 1a-iii? *

Copy and paste relevant sections from manuscript title (include quotes in quotation marks "like this" to indicate direct quotes from your manuscript), or elaborate on this item by providing additional information not in the ms, or briefly explain why the item is not applicable/relevant for your study

"Smoking Cessation"

1b) ABSTRACT: Structured summary of trial design, methods, results, and conclusions

NPT extension: Description of experimental treatment, comparator, care providers, centers, and blinding status.

1b-i) Key features/functionalities/components of the intervention and comparator in the METHODS section of the ABSTRACT

Mention key features/functionalities/components of the intervention and comparator in the abstract. If possible, also mention theories and principles used for designing the site. Keep in mind the needs of systematic reviewers and indexers by including important synonyms. (Note: Only report in the abstract what the main paper is reporting. If this information is missing from the main body of text, consider adding it)

	1	2	3	4	5	
subitem not at all important	○	○	○	○	◉	essential

Você está editando sua resposta. O compartilhamento deste URL permitirá que outras pessoas também a editem.

INSERIR UMA NOVA RESPOSTA

Does your paper address subitem 1b-i? *

Copy and paste relevant sections from the manuscript abstract (include quotes in quotation marks "like this" to indicate direct quotes from your manuscript), or elaborate on this item by providing additional information not in the ms, or briefly explain why the item is not applicable/relevant for your study

"comparing smoking cessation rates", "an intervention group (IG) receiving psychopharmacological treatment and using a mobile app providing artificial intelligence generated and tailored smoking cessation support messages"

1b-ii) Level of human involvement in the METHODS section of the ABSTRACT

Clarify the level of human involvement in the abstract, e.g., use phrases like “fully automated” vs. “therapist/nurse/care provider/physician-assisted” (mention number and expertise of providers involved, if any). (Note: Only report in the abstract what the main paper is reporting. If this information is missing from the main body of text, consider adding it)

	1	2	3	4	5	
subitem not at all important	○	○	○	●	○	essential

Does your paper address subitem 1b-ii?

Copy and paste relevant sections from the manuscript abstract (include quotes in quotation marks "like this" to indicate direct quotes from your manuscript), or elaborate on this item by providing additional information not in the ms, or briefly explain why the item is not applicable/relevant for your study

"artificial intelligence-generated and tailored smoking cessation support messages"

Você está editando sua resposta. O compartilhamento deste URL permitirá que outras pessoas também a editem.

INSERIR UMA NOVA RESPOSTA

1b-iii) Open vs. closed, web-based (self-assessment) vs. face-to-face assessments in the METHODS section of the ABSTRACT

Mention how participants were recruited (online vs. offline), e.g., from an open access website or from a clinic or a closed online user group (closed usergroup trial), and clarify if this was a purely web-based trial, or there were face-to-face components (as part of the intervention or for assessment). Clearly say if outcomes were self-assessed through questionnaires (as common in web-based trials). Note: In traditional offline trials, an open trial (open-label trial) is a type of clinical trial in which both the researchers and participants know which treatment is being administered. To avoid confusion, use “blinded” or “unblinded” to indicated the level of blinding instead of “open”, as “open” in web-based trials usually refers to “open access” (i.e. participants can self-enrol). (Note: Only report in the abstract what the main paper is reporting. If this information is missing from the main body of text, consider adding it)

	1	2	3	4	5	
subitem not at all important	○	○	○	○	◉	essential

Does your paper address subitem 1b-iii?

Copy and paste relevant sections from the manuscript abstract (include quotes in quotation marks "like this" to indicate direct quotes from your manuscript), or elaborate on this item by providing additional information not in the ms, or briefly explain why the item is not applicable/relevant for your study

"A 12-month, randomized, open-label, parallel-group trial"; "Participants were blinded to this allocation, as those in the IG were told that the provided mobile app was part of usual care. Participants in the CG were not informed about the existence of the app and did not have access to it."

1b-iv) RESULTS section in abstract must contain use data

Report number of participants enrolled/assessed in each group, the use/uptake of the intervention (e.g., attrition/adherence metrics, use over time, number of logins etc.), in addition to primary/secondary outcomes. (Note: Only report in the abstract what the main paper is reporting. If this information is missing from the main body of text, consider adding it)

	1	2	3	4	5	
subitem not at all important	○	○	○	○	◉	essential

Você está editando sua resposta. O compartilhamento deste URL permitirá que outras pessoas também a editem.

INSERIR UMA NOVA RESPOSTA

Does your paper address subitem 1b-iv?

Copy and paste relevant sections from the manuscript abstract (include quotes in quotation marks "like this" to indicate direct quotes from your manuscript), or elaborate on this item by providing additional information not in the ms, or briefly explain why the item is not applicable/relevant for your study

Yes, number of participants enrolled/assessed in each group, as well engagement and system appreciation are presented in the methods and results sections.

1b-v) CONCLUSIONS/DISCUSSION in abstract for negative trials

Conclusions/Discussions in abstract for negative trials: Discuss the primary outcome - if the trial is negative (primary outcome not changed), and the intervention was not used, discuss whether negative results are attributable to lack of uptake and discuss reasons. (Note: Only report in the abstract what the main paper is reporting. If this information is missing from the main body of text, consider adding it)

	1	2	3	4	5	
subitem not at all important	(●)	○	○	○	○	essential

Does your paper address subitem 1b-v?

Copy and paste relevant sections from the manuscript abstract (include quotes in quotation marks "like this" to indicate direct quotes from your manuscript), or elaborate on this item by providing additional information not in the ms, or briefly explain why the item is not applicable/relevant for your study

Not applicable, as this is a possitive trial.

INTRODUCTION

2a) In INTRODUCTION: Scientific background and explanation of rationale

Você está editando sua resposta. O compartilhamento deste URL permitirá que outras pessoas também a editem.

INSERIR UMA NOVA RESPOSTA

2a-i) Problem and the type of system/solution

Describe the problem and the type of system/solution that is object of the study: intended as stand-alone intervention vs. incorporated in broader health care program? Intended for a particular patient population? Goals of the intervention, e.g., being more cost-effective to other interventions, replace or complement other solutions? (Note: Details about the intervention are provided in "Methods" under 5)

	1	2	3	4	5	
subitem not at all important	○	○	○	○	◉	essential

Does your paper address subitem 2a-i? *

Copy and paste relevant sections from the manuscript (include quotes in quotation marks "like this" to indicate direct quotes from your manuscript), or elaborate on this item by providing additional information not in the ms, or briefly explain why the item is not applicable/relevant for your study

Problem: "Despite the proven efficacy of these treatments, a meaningful number of smokers fail to stop smoking, and many factors influence their success, including the motivation to stop and continuous support."
Solution: "The experimental intervention focused on providing ubiquitous tailored support to patients willing to stop smoking through a digital therapeutic mobile app solution." This is further described in the same section.

2a-ii) Scientific background, rationale: What is known about the (type of) system

Scientific background, rationale: What is known about the (type of) system that is the object of the study (be sure to discuss the use of similar systems for other conditions/diagnoses, if appropiate), motivation for the study, i.e. what are the reasons for and what is the context for this specific study, from which stakeholder viewpoint is the study performed, potential impact of findings [2]. Briefly justify the choice of the comparator.

	1	2	3	4	5	
subitem not at all important	○	○	○	○	◉	essential

Você está editando sua resposta. O compartilhamento deste URL permitirá que outras pessoas também a editem. INSERIR UMA NOVA RESPOSTA

Does your paper address subitem 2a-ii? *

Copy and paste relevant sections from the manuscript (include quotes in quotation marks "like this" to indicate direct quotes from your manuscript), or elaborate on this item by providing additional information not in the ms, or briefly explain why the item is not applicable/relevant for your study

Yes. It's all included in the Introduction section.

2b) In INTRODUCTION: Specific objectives or hypotheses

Does your paper address CONSORT subitem 2b? *

Copy and paste relevant sections from the manuscript (include quotes in quotation marks "like this" to indicate direct quotes from your manuscript), or elaborate on this item by providing additional information not in the ms, or briefly explain why the item is not applicable/relevant for your study

"The main objective of this So-Lo-Mo study was to compare usual psychopharmacological therapy (control group, CG) alone and alongside the aforementioned digital solution (intervention group, IG) for smoking cessation. The secondary objectives were to analyze health-related quality of life (HRQoL) and monitor healthy lifestyle and physical exercise habits. Complementarily, we assessed the impact of the AI-generated motivational messages on smoking cessation outcomes in the IG."

METHODS

3a) Description of trial design (such as parallel, factorial) including allocation ratio

Você está editando sua resposta. O compartilhamento deste URL permitirá que outras pessoas também a editem.

INSERIR UMA NOVA RESPOSTA

Does your paper address CONSORT subitem 3a? *

Copy and paste relevant sections from the manuscript (include quotes in quotation marks "like this" to indicate direct quotes from your manuscript), or elaborate on this item by providing additional information not in the ms, or briefly explain why the item is not applicable/relevant for your study

"This 12-month, randomized, open-label, parallel-group trial was performed at the Smoking Cessation Unit of Virgen del RocÃo University Hospital in Seville (Spain) between October 24, 2016, and October 24, 2018, and it complied with the Declaration of Helsinki and Good Clinical Practice Guidelines. The recruitment period closed on October 23, 2017. The local ethics committee approved the study protocol, and written informed consent was obtained from each participant prior to inclusion. The clinical study design (NCT03553173) and technical study design (NCT03206619) have been published previously [23,24]."

3b) Important changes to methods after trial commencement (such as eligibility criteria), with reasons

Does your paper address CONSORT subitem 3b? *

Copy and paste relevant sections from the manuscript (include quotes in quotation marks "like this" to indicate direct quotes from your manuscript), or elaborate on this item by providing additional information not in the ms, or briefly explain why the item is not applicable/relevant for your study

Not applicable, there were no important changes in methods after trial commencement.

3b-i) Bug fixes, Downtimes, Content Changes

Bug fixes, Downtimes, Content Changes: ehealth systems are often dynamic systems. A description of changes to methods therefore also includes important changes made on the intervention or comparator during the trial (e.g., major bug fixes or changes in the functionality or content) (5-iii) and other “unexpected events” that may have influenced study design such as staff changes, system failures/downtimes, etc. [2].

	1	2	3	4	5	
subitem not at all important	○	●	○	○	○	essential

Você está editando sua resposta. O compartilhamento deste URL permitirá que outras pessoas também a editem.

INSERIR UMA NOVA RESPOSTA

Does your paper address subitem 3b-i?

Copy and paste relevant sections from the manuscript (include quotes in quotation marks "like this" to indicate direct quotes from your manuscript), or elaborate on this item by providing additional information not in the ms, or briefly explain why the item is not applicable/relevant for your study

The eHealth system didn't experience any bug, downtime and/or content changes during the trial procedure.

4a) Eligibility criteria for participants

Does your paper address CONSORT subitem 4a? *

Copy and paste relevant sections from the manuscript (include quotes in quotation marks "like this" to indicate direct quotes from your manuscript), or elaborate on this item by providing additional information not in the ms, or briefly explain why the item is not applicable/relevant for your study

The inclusion criteria were as follows: (1) age over 18 years and desire to stop smoking; (2) owning an Android smartphone (as the mobile app was only available for Android devices owing to time and resource constraints in the development phase of this study and Android phones were more likely to be owned by the target population owing to their lower entry price as compared with iPhones); and (3) ability to interact with the smartphone. Smartphone literacy was assessed by asking the participants if they commonly use other text exchange smartphone apps, such as Mail, SMS, and WhatsApp. The only exclusion criterion was any previous adverse effect related to the present pharmacological treatment.

4a-i) Computer / Internet literacy

Computer / Internet literacy is often an implicit “de facto” eligibility criterion - this should be explicitly clarified.

	1	2	3	4	5	
subitem not at all important	○	○	○	◉	○	essential

Você está editando sua resposta. O compartilhamento deste URL permitirá que outras pessoas também a editem.

INSERIR UMA NOVA RESPOSTA

Does your paper address subitem 4a-i?

Copy and paste relevant sections from the manuscript (include quotes in quotation marks "like this" to indicate direct quotes from your manuscript), or elaborate on this item by providing additional information not in the ms, or briefly explain why the item is not applicable/relevant for your study

"ability to interact with the smartphone. Smartphone literacy was assessed by asking the participants if they commonly use other text exchange smartphone apps, such as Mail, SMS, and WhatsApp."

4a-ii) Open vs. closed, web-based vs. face-to-face assessments:

Open vs. closed, web-based vs. face-to-face assessments: Mention how participants were recruited (online vs. offline), e.g., from an open access website or from a clinic, and clarify if this was a purely web-based trial, or there were face-to-face components (as part of the intervention or for assessment), i.e., to what degree got the study team to know the participant. In online-only trials, clarify if participants were quasi-anonymous and whether having multiple identities was possible or whether technical or logistical measures (e.g., cookies, email confirmation, phone calls) were used to detect/prevent these.

	1	2	3	4	5	
subitem not at all important	○	○	○	○	◉	essential

Does your paper address subitem 4a-ii? *

Copy and paste relevant sections from the manuscript (include quotes in quotation marks "like this" to indicate direct quotes from your manuscript), or elaborate on this item by providing additional information not in the ms, or briefly explain why the item is not applicable/relevant for your study

"Smokers were recruited during routine visits to our outpatient clinic."

4a-iii) Information giving during recruitment

Information given during recruitment. Specify how participants were briefed for recruitment and in the informed consent procedures (e.g., publish the informed consent documentation as appendix, see also item X26), as this information may have an effect on user self-selection, user expectation and may also bias results.

	1	2	3	4	5	
subitem not at all important	○	◉	○	○	○	essential

Você está editando sua resposta. O compartilhamento deste URL permitirá que outras pessoas também a editem.

INSERIR UMA NOVA RESPOSTA

Does your paper address subitem 4a-iii?

Copy and paste relevant sections from the manuscript (include quotes in quotation marks "like this" to indicate direct quotes from your manuscript), or elaborate on this item by providing additional information not in the ms, or briefly explain why the item is not applicable/relevant for your study

Information given during recruitment is not included in the paper.

4b) Settings and locations where the data were collected

Does your paper address CONSORT subitem 4b? *

Copy and paste relevant sections from the manuscript (include quotes in quotation marks "like this" to indicate direct quotes from your manuscript), or elaborate on this item by providing additional information not in the ms, or briefly explain why the item is not applicable/relevant for your study

"Smoking Cessation Unit of Virgen del Rocio University Hospital in Seville (Spain)"

4b-i) Report if outcomes were (self-)assessed through online questionnaires

Clearly report if outcomes were (self-)assessed through online questionnaires (as common in web-based trials) or otherwise.

	1	2	3	4	5	
subitem not at all important	(●)	○	○	○	○	essential

Does your paper address subitem 4b-i? *

Copy and paste relevant sections from the manuscript (include quotes in quotation marks "like this" to indicate direct quotes from your manuscript), or elaborate on this item by providing additional information not in the ms, or briefly explain why the item is not applicable/relevant for your study

"The main clinical outcome was the 1-year smoking abstinence rate measured by exhaled carbon monoxide (CO), which was assessed using a CO tester (Micro+ Smokerlyzer; CoVita, Santa Barbara, California, USA) and urine cotinine tests."

Você está editando sua resposta. O compartilhamento deste URL permitirá que outras pessoas também a editem.

INSERIR UMA NOVA RESPOSTA

4b-ii) Report how institutional affiliations are displayed

Report how institutional affiliations are displayed to potential participants [on ehealth media], as affiliations with prestigious hospitals or universities may affect volunteer rates, use, and reactions with regards to an intervention.(Not a required item – describe only if this may bias results)

	1	2	3	4	5	
subitem not at all important	◉	○	○	○	○	essential

Does your paper address subitem 4b-ii?

Copy and paste relevant sections from the manuscript (include quotes in quotation marks "like this" to indicate direct quotes from your manuscript), or elaborate on this item by providing additional information not in the ms, or briefly explain why the item is not applicable/relevant for your study

Not applicable, as this is not the case for this study. Participants were recruited on a routine care basis, not through any kind of advertisement.

5) The interventions for each group with sufficient details to allow replication, including how and when they were actually administered

5-i) Mention names, credential, affiliations of the developers, sponsors, and owners

Mention names, credential, affiliations of the developers, sponsors, and owners [6] (if authors/evaluators are owners or developer of the software, this needs to be declared in a "Conflict of interest" section or mentioned elsewhere in the manuscript).

	1	2	3	4	5	
subitem not at all important	○	○	○	◉	○	essential

Você está editando sua resposta. O compartilhamento deste URL permitirá que outras pessoas também a editem.

INSERIR UMA NOVA RESPOSTA

Does your paper address subitem 5-i?

Copy and paste relevant sections from the manuscript (include quotes in quotation marks "like this" to indicate direct quotes from your manuscript), or elaborate on this item by providing additional information not in the ms, or briefly explain why the item is not applicable/relevant for your study

"The digital therapeutic solution used in the So-Lo-Mo study was jointly developed by Salumedia Tecnologias SLU, the Aristotle University of Thessaloniki, Servicio Andaluz de Salud, and Universidad de Sevilla based on the SmokeFreeBrain project. We acknowledge Prof Hein de Vries and Dr Francine Schneider who provided guidance in the data analysis of the technical outcomes. This research was funded by the H2020 European Commission research and innovation program (grant agreement 681120) as part of the SmokeFreeBrain project (www.smokefreebrain.eu)."

5-ii) Describe the history/development process

Describe the history/development process of the application and previous formative evaluations (e.g., focus groups, usability testing), as these will have an impact on adoption/use rates and help with interpreting results.

	1	2	3	4	5	
subitem not at all important	○	○	○	◉	○	essential

Does your paper address subitem 5-ii?

Copy and paste relevant sections from the manuscript (include quotes in quotation marks "like this" to indicate direct quotes from your manuscript), or elaborate on this item by providing additional information not in the ms, or briefly explain why the item is not applicable/relevant for your study

"Specifically, the AI system used to tailor motivational messages is a hybrid HRS, whose design has been presented by Hors-Fraile et al [20]."; "The motivational messages were designed after conducting semistructured interviews involving two smoking cessation experts (a pulmonologist and a psychologist)."

Você está editando sua resposta. O compartilhamento deste URL permitirá que outras pessoas também a editem.

INSERIR UMA NOVA RESPOSTA

5-iii) Revisions and updating

Revisions and updating. Clearly mention the date and/or version number of the application/intervention (and comparator, if applicable) evaluated, or describe whether the intervention underwent major changes during the evaluation process, or whether the development and/or content was "frozen" during the trial. Describe dynamic components such as news feeds or changing content which may have an impact on the replicability of the intervention (for unexpected events see item 3b).

	1	2	3	4	5	
subitem not at all important	○	○	○	◉	○	essential

Does your paper address subitem 5-iii?

Copy and paste relevant sections from the manuscript (include quotes in quotation marks "like this" to indicate direct quotes from your manuscript), or elaborate on this item by providing additional information not in the ms, or briefly explain why the item is not applicable/relevant for your study

"between October 24, 2016, and October 24, 2018"

5-iv) Quality assurance methods

Provide information on quality assurance methods to ensure accuracy and quality of information provided [1], if applicable.

	1	2	3	4	5	
subitem not at all important	○	○	○	○	◉	essential

Does your paper address subitem 5-iv?

Copy and paste relevant sections from the manuscript (include quotes in quotation marks "like this" to indicate direct quotes from your manuscript), or elaborate on this item by providing additional information not in the ms, or briefly explain why the item is not applicable/relevant for your study

"The motivational messages were designed after conducting semistructured interviews involving two smoking cessation experts (a pulmonologist and a psychologist). A total of 150 different messages were written for each topic by a health communication and health promotion PhD candidate, and they were validated by the two smoking cessation experts."

Você está editando sua resposta. O compartilhamento deste URL permitirá que outras pessoas também a editem.

INSERIR UMA NOVA RESPOSTA

5-v) Ensure replicability by publishing the source code, and/or providing screenshots/screen-capture video, and/or providing flowcharts of the algorithms used

Ensure replicability by publishing the source code, and/or providing screenshots/screen-capture video, and/or providing flowcharts of the algorithms used. Replicability (i.e., other researchers should in principle be able to replicate the study) is a hallmark of scientific reporting.

	1	2	3	4	5	
subitem not at all important	○	◉	○	○	○	essential

Does your paper address subitem 5-v?

Copy and paste relevant sections from the manuscript (include quotes in quotation marks "like this" to indicate direct quotes from your manuscript), or elaborate on this item by providing additional information not in the ms, or briefly explain why the item is not applicable/relevant for your study

It is not possible for the authors to publish the source code as the IPR of this mHealth intervention are protected. Algorithms used are already published and referenced in the document: "Specifically, the AI system used to tailor motivational messages is a hybrid HRS, whose design has been presented by Hors-Fraile et al [20]."

5-vi) Digital preservation

Digital preservation: Provide the URL of the application, but as the intervention is likely to change or disappear over the course of the years; also make sure the intervention is archived (Internet Archive, webcitation.org, and/or publishing the source code or screenshots/videos alongside the article). As pages behind login screens cannot be archived, consider creating demo pages which are accessible without login.

	1	2	3	4	5	
subitem not at all important	○	◉	○	○	○	essential

Você está editando sua resposta. O compartilhamento deste URL permitirá que outras pessoas também a editem.

INSERIR UMA NOVA RESPOSTA

Does your paper address subitem 5-vi?

Copy and paste relevant sections from the manuscript (include quotes in quotation marks "like this" to indicate direct quotes from your manuscript), or elaborate on this item by providing additional information not in the ms, or briefly explain why the item is not applicable/relevant for your study

This information is not available in the paper itself. However, you can acces the app here (this is only the front-end, back-end is not available at the moment): https://apkpure.com/kr/libre-de-humos/com.salumedia.smokefreebrain, and a video here: https://www.youtube.com/watch?v=IZCEw9jAXuc

5-vii) Access

Access: Describe how participants accessed the application, in what setting/context, if they had to pay (or were paid) or not, whether they had to be a member of specific group. If known, describe how participants obtained "access to the platform and Internet" [1]. To ensure access for editors/reviewers/readers, consider to provide a "backdoor" login account or demo mode for reviewers/readers to explore the application (also important for archiving purposes, see vi).

	1	2	3	4	5	
subitem not at all important	○	○	○	○	◉	essential

Does your paper address subitem 5-vii? *

Copy and paste relevant sections from the manuscript (include quotes in quotation marks "like this" to indicate direct quotes from your manuscript), or elaborate on this item by providing additional information not in the ms, or briefly explain why the item is not applicable/relevant for your study

"all participants received their assigned treatments free of charge. [...] IG participants (n=120) received psychopharmacological therapy and used the digital therapeutic solution."

Você está editando sua resposta. O compartilhamento deste URL permitirá que outras pessoas também a editem.

INSERIR UMA NOVA RESPOSTA

5-viii) Mode of delivery, features/functionalities/components of the intervention and comparator, and the theoretical framework

Describe mode of delivery, features/functionalities/components of the intervention and comparator, and the theoretical framework [6] used to design them (instructional strategy [1], behaviour change techniques, persuasive features, etc., see e.g., [7, 8] for terminology). This includes an in-depth description of the content (including where it is coming from and who developed it) [1]," whether [and how] it is tailored to individual circumstances and allows users to track their progress and receive feedback" [6]. This also includes a description of communication delivery channels and – if computer-mediated communication is a component – whether communication was synchronous or asynchronous [6]. It also includes information on presentation strategies [1], including page design principles, average amount of text on pages, presence of hyperlinks to other resources, etc. [1].

	1	2	3	4	5	
subitem not at all important	○	○	○	○	◉	essential

Does your paper address subitem 5-viii? *

Copy and paste relevant sections from the manuscript (include quotes in quotation marks "like this" to indicate direct quotes from your manuscript), or elaborate on this item by providing additional information not in the ms, or briefly explain why the item is not applicable/relevant for your study

"The app utilized behavioral techniques by sending personalized motivational messages generated using AI with the intent to achieve better smoking cessation rates by improving program adherence and abstinence rates [20]." See figure 2 for further information.

5-ix) Describe use parameters

Describe use parameters (e.g., intended "doses" and optimal timing for use). Clarify what instructions or recommendations were given to the user, e.g., regarding timing, frequency, heaviness of use, if any, or was the intervention used ad libitum.

	1	2	3	4	5	
subitem not at all important	○	○	○	○	◉	essential

Você está editando sua resposta. O compartilhamento deste URL permitirá que outras pessoas também a editem.

INSERIR UMA NOVA RESPOSTA

Does your paper address subitem 5-ix?

Copy and paste relevant sections from the manuscript (include quotes in quotation marks "like this" to indicate direct quotes from your manuscript), or elaborate on this item by providing additional information not in the ms, or briefly explain why the item is not applicable/relevant for your study

"Time to Open Motivational Messages: This pertained to the arithmetic mean of the elapsed time between the message being sent and opened in a 30-day period."; "Engagement With the System: To measure system engagement, we included a time stamp in each sent message and compared it with the time stamp sent by the app to the server when the user opened the given message."

5-x) Clarify the level of human involvement

Clarify the level of human involvement (care providers or health professionals, also technical assistance) in the e-intervention or as co-intervention (detail number and expertise of professionals involved, if any, as well as “type of assistance offered, the timing and frequency of the support, how it is initiated, and the medium by which the assistance is delivered”. It may be necessary to distinguish between the level of human involvement required for the trial, and the level of human involvement required for a routine application outside of a RCT setting (discuss under item 21 – generalizability).

	1	2	3	4	5	
subitem not at all important	○	○	○	○	◉	essential

Does your paper address subitem 5-x?

Copy and paste relevant sections from the manuscript (include quotes in quotation marks "like this" to indicate direct quotes from your manuscript), or elaborate on this item by providing additional information not in the ms, or briefly explain why the item is not applicable/relevant for your study

"The motivational messages were designed after conducting semistructured interviews involving two smoking cessation experts (a pulmonologist and a psychologist). A total of 150 different messages were written for each topic by a health communication and health promotion PhD candidate, and they were validated by the two smoking cessation experts."

Você está editando sua resposta. O compartilhamento deste URL permitirá que outras pessoas também a editem.

INSERIR UMA NOVA RESPOSTA

5-xi) Report any prompts/reminders used

Report any prompts/reminders used: Clarify if there were prompts (letters, emails, phone calls, SMS) to use the application, what triggered them, frequency etc. It may be necessary to distinguish between the level of prompts/reminders required for the trial, and the level of prompts/reminders for a routine application outside of a RCT setting (discuss under item 21 – generalizability).

	1	2	3	4	5	
subitem not at all important	○	○	○	○	◉	essential

Does your paper address subitem 5-xi? *

Copy and paste relevant sections from the manuscript (include quotes in quotation marks "like this" to indicate direct quotes from your manuscript), or elaborate on this item by providing additional information not in the ms, or briefly explain why the item is not applicable/relevant for your study

"The mobile app was connected with an AI system designed to learn from patient interests through their interactions with the app to dynamically (1) determine, personalize, and send motivational messages to support smoking cessation; (2) schedule the message delivery frequency according to the transtheoretical model of behavioral change [19]; and (3) calculate the most convenient time to send a motivational message to support smoking cessation for each patient. Specifically, the AI system used to tailor motivational messages is a hybrid HRS, whose design has been presented by Hors-Fraile et al [20]."

5-xii) Describe any co-interventions (incl. training/support)

Describe any co-interventions (incl. training/support): Clearly state any interventions that are provided in addition to the targeted eHealth intervention, as ehealth intervention may not be designed as stand-alone intervention. This includes training sessions and support [1]. It may be necessary to distinguish between the level of training required for the trial, and the level of training for a routine application outside of a RCT setting (discuss under item 21 – generalizability.

	1	2	3	4	5	
subitem not at all important	◉	○	○	○	○	essential

Você está editando sua resposta. O compartilhamento deste URL permitirá que outras pessoas também a editem.

INSERIR UMA NOVA RESPOSTA

Does your paper address subitem 5-xii? *

Copy and paste relevant sections from the manuscript (include quotes in quotation marks "like this" to indicate direct quotes from your manuscript), or elaborate on this item by providing additional information not in the ms, or briefly explain why the item is not applicable/relevant for your study

There were no co-intervention, the mHealth solution included a step-by-step guide for the users.

6a) Completely defined pre-specified primary and secondary outcome measures, including how and when they were assessed

Does your paper address CONSORT subitem 6a? *

Copy and paste relevant sections from the manuscript (include quotes in quotation marks "like this" to indicate direct quotes from your manuscript), or elaborate on this item by providing additional information not in the ms, or briefly explain why the item is not applicable/relevant for your study

"The clinical study design (NCT03553173) [...] have been published previously [23,24]."

6a-i) Online questionnaires: describe if they were validated for online use and apply CHERRIES items to describe how the questionnaires were designed/deployed

If outcomes were obtained through online questionnaires, describe if they were validated for online use and apply CHERRIES items to describe how the questionnaires were designed/deployed [9].

	1	2	3	4	5	
subitem not at all important	(•)	( )	( )	( )	( )	essential

Does your paper address subitem 6a-i?

Copy and paste relevant sections from manuscript text

Online questionnaires were not used as part of this work.

Você está editando sua resposta. O compartilhamento deste URL permitirá que outras pessoas também a editem.

INSERIR UMA NOVA RESPOSTA

6a-ii) Describe whether and how “use” (including intensity of use/dosage) was defined/measured/monitored

Describe whether and how “use” (including intensity of use/dosage) was defined/measured/monitored (logins, logfile analysis, etc.). Use/adoption metrics are important process outcomes that should be reported in any ehealth trial.

	1	2	3	4	5	
subitem not at all important	○	○	○	◉	○	essential

Does your paper address subitem 6a-ii?

Copy and paste relevant sections from manuscript text

"Time to Open Motivational Messages: This pertained to the arithmetic mean of the elapsed time between the message being sent and opened in a 30-day period."; "Engagement With the System: To measure system engagement, we included a time stamp in each sent message and compared it with the time stamp sent by the app to the server when the user opened the given message."

6a-iii) Describe whether, how, and when qualitative feedback from participants was obtained

Describe whether, how, and when qualitative feedback from participants was obtained (e.g., through emails, feedback forms, interviews, focus groups).

	1	2	3	4	5	
subitem not at all important	○	○	○	○	◉	essential

Does your paper address subitem 6a-iii?

Copy and paste relevant sections from manuscript text

"Subjective Quality of the System System quality was determined by the answers in an anonymized five-level Likert-type appreciation questionnaire [24], making it impossible to link the answers to the individual participants."

Você está editando sua resposta. O compartilhamento deste URL permitirá que outras pessoas também a editem. INSERIR UMA NOVA RESPOSTA

6b) Any changes to trial outcomes after the trial commenced, with reasons

Does your paper address CONSORT subitem 6b? *

Copy and paste relevant sections from the manuscript (include quotes in quotation marks "like this" to indicate direct quotes from your manuscript), or elaborate on this item by providing additional information not in the ms, or briefly explain why the item is not applicable/relevant for your study

No changes at all after trial commenced.

7a) How sample size was determined

NPT: When applicable, details of whether and how the clustering by care provides or centers was addressed

7a-i) Describe whether and how expected attrition was taken into account when calculating the sample size

Describe whether and how expected attrition was taken into account when calculating the sample size.

	1	2	3	4	5	
subitem not at all important	○	○	○	○	◉	essential

Does your paper address subitem 7a-i?

Copy and paste relevant sections from manuscript title (include quotes in quotation marks "like this" to indicate direct quotes from your manuscript), or elaborate on this item by providing additional information not in the ms, or briefly explain why the item is not applicable/relevant for your study

"Power Calculation and Recruitment: A sample size of 236 was calculated during the study design phase, according to the following parameters: CI, 95%; statistical power, 80%; CG success rate, 35%; IG success rate, 55%; and expected dropout rate, 20%. A total of 240 participants were recruited and randomized for the stratified analysis."

7b) When applicable, explanation of any interim analyses and stopping guidelines

Você está editando sua resposta. O compartilhamento deste URL permitirá que outras pessoas também a editem.

INSERIR UMA NOVA RESPOSTA

Does your paper address CONSORT subitem 7b? *

Copy and paste relevant sections from the manuscript (include quotes in quotation marks "like this" to indicate direct quotes from your manuscript), or elaborate on this item by providing additional information not in the ms, or briefly explain why the item is not applicable/relevant for your study

No stopping guidelines were defined. An interim analysis at 6 months was performed to commit with a deliverable related to the funding project.

8a) Method used to generate the random allocation sequence

NPT: When applicable, how care providers were allocated to each trial group

Does your paper address CONSORT subitem 8a? *

Copy and paste relevant sections from the manuscript (include quotes in quotation marks "like this" to indicate direct quotes from your manuscript), or elaborate on this item by providing additional information not in the ms, or briefly explain why the item is not applicable/relevant for your study

"A technician generated a random-group table (n=240) using computer methods and following a 1:1 ratio between groups. Clinicians enrolled the participants and assigned them to the group mentioned in the table according to their enrolment sequence."

8b) Type of randomisation; details of any restriction (such as blocking and block size)

Does your paper address CONSORT subitem 8b? *

Copy and paste relevant sections from the manuscript (include quotes in quotation marks "like this" to indicate direct quotes from your manuscript), or elaborate on this item by providing additional information not in the ms, or briefly explain why the item is not applicable/relevant for your study

"A technician generated a random-group table (n=240) using computer methods and following a 1:1 ratio between groups."

9) Mechanism used to implement the random allocation sequence (such as sequentially numbered containers), describing any steps taken to conceal the

Você está editando sua resposta. O compartilhamento deste URL permitirá que outras pessoas também a editem.

INSERIR UMA NOVA RESPOSTA

Does your paper address CONSORT subitem 9? *

Copy and paste relevant sections from the manuscript (include quotes in quotation marks "like this" to indicate direct quotes from your manuscript), or elaborate on this item by providing additional information not in the ms, or briefly explain why the item is not applicable/relevant for your study

"A technician generated a random-group table (n=240) using computer methods and following a 1:1 ratio between groups. Clinicians enrolled the participants and assigned them to the group mentioned in the table according to their enrolment sequence."

10) Who generated the random allocation sequence, who enrolled participants, and who assigned participants to interventions

Does your paper address CONSORT subitem 10? *

Copy and paste relevant sections from the manuscript (include quotes in quotation marks "like this" to indicate direct quotes from your manuscript), or elaborate on this item by providing additional information not in the ms, or briefly explain why the item is not applicable/relevant for your study

"A technician generated a random-group table (n=240) using computer methods and following a 1:1 ratio between groups. Clinicians enrolled the participants and assigned them to the group mentioned in the table according to their enrolment sequence."

11a) If done, who was blinded after assignment to interventions (for example, participants, care providers, those assessing outcomes) and how

NPT: Whether or not administering co-interventions were blinded to group assignment

11a-i) Specify who was blinded, and who wasn't

Specify who was blinded, and who wasn't. Usually, in web-based trials it is not possible to blind the participants [1, 3] (this should be clearly acknowledged), but it may be possible to blind outcome assessors, those doing data analysis or those administering co-interventions (if any).

	1	2	3	4	5	
subitem not at all important	○	○	○	○	●	essential

Você está editando sua resposta. O compartilhamento deste URL permitirá que outras pessoas também a editem.

INSERIR UMA NOVA RESPOSTA

Does your paper address subitem 11a-i? *

Copy and paste relevant sections from the manuscript (include quotes in quotation marks "like this" to indicate direct quotes from your manuscript), or elaborate on this item by providing additional information not in the ms, or briefly explain why the item is not applicable/relevant for your study

"Participants were blinded to this allocation, as those in the IG were told that the provided mobile app was part of usual care. Participants in the CG were not informed about the existence of the app and did not have access to it."

11a-ii) Discuss e.g., whether participants knew which intervention was the “intervention of interest” and which one was the “comparator”

Informed consent procedures (4a-ii) can create biases and certain expectations - discuss e.g., whether participants knew which intervention was the “intervention of interest” and which one was the “comparator”.

	1	2	3	4	5	
subitem not at all important	○	○	○	○	◉	essential

Does your paper address subitem 11a-ii?

Copy and paste relevant sections from the manuscript (include quotes in quotation marks "like this" to indicate direct quotes from your manuscript), or elaborate on this item by providing additional information not in the ms, or briefly explain why the item is not applicable/relevant for your study

"Participants were blinded to this allocation, as those in the IG were told that the provided mobile app was part of usual care. Participants in the CG were not informed about the existence of the app and did not have access to it."

11b) If relevant, description of the similarity of interventions

(this item is usually not relevant for ehealth trials as it refers to similarity of a placebo or sham intervention to a active medication/intervention)

Você está editando sua resposta. O compartilhamento deste URL permitirá que outras pessoas também a editem.

INSERIR UMA NOVA RESPOSTA

Does your paper address CONSORT subitem 11b? *

Copy and paste relevant sections from the manuscript (include quotes in quotation marks "like this" to indicate direct quotes from your manuscript), or elaborate on this item by providing additional information not in the ms, or briefly explain why the item is not applicable/relevant for your study

"Psychopharmacological therapy was provided to both CG and IG participants. CG participants (n=120) received psychopharmacological therapy alone, whereas IG participants (n=120) received psychopharmacological therapy and used the digital therapeutic solution."

12a) Statistical methods used to compare groups for primary and secondary outcomes

NPT: When applicable, details of whether and how the clustering by care providers or centers was addressed

Does your paper address CONSORT subitem 12a? *

Copy and paste relevant sections from the manuscript (include quotes in quotation marks "like this" to indicate direct quotes from your manuscript), or elaborate on this item by providing additional information not in the ms, or briefly explain why the item is not applicable/relevant for your study

"Descriptive analyses of participant characteristics according to absolute and relative frequencies for qualitative variables and mean (SD) for quantitative variables were conducted. [...] Regarding incomplete data analyses, homoscedasticity tests were conducted among groups of cases with identical missing data patterns to evaluate whether data were missing completely at random [35]. A multinomial regression analysis was performed to assess the variables influencing the probability of a participant dropping out as compared with the probability of the treatment being effective. A no efficacy category was set as the reference category. As multinomial model effects are relative to the reference category, to assess whether the explicative variable effects were different between the dropout and efficacy categories, the same model was adjusted using the dropout category as a reference. Consequently, whether the variable effects were different between the dropout and efficacy categories was assessed. The relative risk ratio (RRR) with 95% CI was used for each model."

Você está editando sua resposta. O compartilhamento deste URL permitirá que outras pessoas também a editem.

INSERIR UMA NOVA RESPOSTA

12a-i) Imputation techniques to deal with attrition / missing values

Imputation techniques to deal with attrition / missing values: Not all participants will use the intervention/comparator as intended and attrition is typically high in ehealth trials. Specify how participants who did not use the application or dropped out from the trial were treated in the statistical analysis (a complete case analysis is strongly discouraged, and simple imputation techniques such as LOCF may also be problematic [4]).

	1	2	3	4	5	
subitem not at all important	○	○	○	○	◉	essential

Does your paper address subitem 12a-i? *

Copy and paste relevant sections from the manuscript (include quotes in quotation marks "like this" to indicate direct quotes from your manuscript), or elaborate on this item by providing additional information not in the ms, or briefly explain why the item is not applicable/relevant for your study

"Lost data analysis and multinomial logistic models showed different patterns in participants who dropped out as compared with the patterns in those who completed the study, regardless of treatment efficacy. Therefore, smoking abstinence at 1 year was analyzed using logistic regression models on a per-protocol basis and intention-to-treat basis, and the effect measures were the OR and 95% CI."

12b) Methods for additional analyses, such as subgroup analyses and adjusted analyses

Você está editando sua resposta. O compartilhamento deste URL permitirá que outras pessoas também a editem.

INSERIR UMA NOVA RESPOSTA

Does your paper address CONSORT subitem 12b? *

Copy and paste relevant sections from the manuscript (include quotes in quotation marks "like this" to indicate direct quotes from your manuscript), or elaborate on this item by providing additional information not in the ms, or briefly explain why the item is not applicable/relevant for your study

"For the selection of logistic regression and multinomial logistic models in per-protocol (n=94) analysis and intention-to-treat (n=240) analysis, a two-stage strategy was adopted. In the first step, the variable importance was quantified using Random Forest [36] with the mean decrease in accuracy as the score. Variables with a score above 0.5 were included in Akaike information criteria based stepwise selection strategy. The following two restrictions were applied to the final models selected: (1) absence of a pattern in model residuals and (2) variables with a generalized variance inflation factor [37] above 5 were not allowed in order to avoid collinearity. To determine whether the HRS metrics had an impact on the clinical outcomes, we divided IG participants in cessation and no cessation subgroups at 12 months of the intervention. Thereafter, we conducted t tests for the 12-month results of precision, time to rate messages, and engagement, analyzing each of these two subgroups."

X26) REB/IRB Approval and Ethical Considerations [recommended as subheading under "Methods"] (not a CONSORT item)

X26-i) Comment on ethics committee approval

	1	2	3	4	5	
subitem not at all important	○	○	○	○	◉	essential

Does your paper address subitem X26-i?

Copy and paste relevant sections from the manuscript (include quotes in quotation marks "like this" to indicate direct quotes from your manuscript), or elaborate on this item by providing additional information not in the ms, or briefly explain why the item is not applicable/relevant for your study

"The local ethics committee approved the study protocol, and written informed consent was obtained from each participant prior to inclusion. The clinical study design (NCT03553173) and technical study design (NCT03206619) have been published previously [23,24]."

Você está editando sua resposta. O compartilhamento deste URL permitirá que outras pessoas também a editem.

INSERIR UMA NOVA RESPOSTA

x26-ii) Outline informed consent procedures

Outline informed consent procedures e.g., if consent was obtained offline or online (how? Checkbox, etc.?), and what information was provided (see 4a-ii). See [6] for some items to be included in informed consent documents.

	1	2	3	4	5	
subitem not at all important	○	○	○	○	◉	essential

Does your paper address subitem X26-ii?

Copy and paste relevant sections from the manuscript (include quotes in quotation marks "like this" to indicate direct quotes from your manuscript), or elaborate on this item by providing additional information not in the ms, or briefly explain why the item is not applicable/relevant for your study

"Written informed consent was obtained from each participant prior to inclusion."

X26-iii) Safety and security procedures

Safety and security procedures, incl. privacy considerations, and any steps taken to reduce the likelihood or detection of harm (e.g., education and training, availability of a hotline)

	1	2	3	4	5	
subitem not at all important	○	○	○	○	◉	essential

Does your paper address subitem X26-iii?

Copy and paste relevant sections from the manuscript (include quotes in quotation marks "like this" to indicate direct quotes from your manuscript), or elaborate on this item by providing additional information not in the ms, or briefly explain why the item is not applicable/relevant for your study

No risk of harm was detected for the mHealth intervention. Regarding pharmacological intervention, safety was monitored in terms of adverse events observed.

[illegible]

Você está editando sua resposta. O compartilhamento deste URL permitirá que outras pessoas também a editem.

INSERIR UMA NOVA RESPOSTA

13a) For each group, the numbers of participants who were randomly assigned, received intended treatment, and were analysed for the primary outcome

NPT: The number of care providers or centers performing the intervention in each group and the number of patients treated by each care provider in each center

Does your paper address CONSORT subitem 13a? *

Copy and paste relevant sections from the manuscript (include quotes in quotation marks "like this" to indicate direct quotes from your manuscript), or elaborate on this item by providing additional information not in the ms, or briefly explain why the item is not applicable/relevant for your study

Yes, this information is presented in Table 1 of the paper.

13b) For each group, losses and exclusions after randomisation, together with reasons

Does your paper address CONSORT subitem 13b? (NOTE: Preferably, this is shown in a CONSORT flow diagram) *

Copy and paste relevant sections from the manuscript (include quotes in quotation marks "like this" to indicate direct quotes from your manuscript), or elaborate on this item by providing additional information not in the ms, or briefly explain why the item is not applicable/relevant for your study

Yes, this information is presented in Figure 1 of the paper.

13b-i) Attrition diagram

Strongly recommended: An attrition diagram (e.g., proportion of participants still logging in or using the intervention/comparator in each group plotted over time, similar to a survival curve) or other figures or tables demonstrating usage/dose/engagement.

	1	2	3	4	5	
subitem not at all important	○	○	○	●	○	essential

Você está editando sua resposta. O compartilhamento deste URL permitirá que outras pessoas também a editem.

INSERIR UMA NOVA RESPOSTA

Does your paper address subitem 13b-i?

Copy and paste relevant sections from the manuscript or cite the figure number if applicable (include quotes in quotation marks "like this" to indicate direct quotes from your manuscript), or elaborate on this item by providing additional information not in the ms, or briefly explain why the item is not applicable/relevant for your study

Yes, this information is presented in Figure 6 of the paper.

14a) Dates defining the periods of recruitment and follow-up

Does your paper address CONSORT subitem 14a? *

Copy and paste relevant sections from the manuscript (include quotes in quotation marks "like this" to indicate direct quotes from your manuscript), or elaborate on this item by providing additional information not in the ms, or briefly explain why the item is not applicable/relevant for your study

"between October 24, 2016, and October 24, 2018"

14a-i) Indicate if critical “secular events” fell into the study period

Indicate if critical “secular events” fell into the study period, e.g., significant changes in Internet resources available or “changes in computer hardware or Internet delivery resources”

	1	2	3	4	5	
subitem not at all important	○	●	○	○	○	essential

Does your paper address subitem 14a-i?

Copy and paste relevant sections from the manuscript (include quotes in quotation marks "like this" to indicate direct quotes from your manuscript), or elaborate on this item by providing additional information not in the ms, or briefly explain why the item is not applicable/relevant for your study

Not applicable, this situation didn't take place in this study.

Você está editando sua resposta. O compartilhamento deste URL permitirá que outras pessoas também a editem.

INSERIR UMA NOVA RESPOSTA

Does your paper address CONSORT subitem 14b? *

Copy and paste relevant sections from the manuscript (include quotes in quotation marks "like this" to indicate direct quotes from your manuscript), or elaborate on this item by providing additional information not in the ms, or briefly explain why the item is not applicable/relevant for your study

Not applicable, the trial ended according to the study protocol.

15) A table showing baseline demographic and clinical characteristics for each group

NPT: When applicable, a description of care providers (case volume, qualification, expertise, etc.) and centers (volume) in each group

Does your paper address CONSORT subitem 15? *

Copy and paste relevant sections from the manuscript (include quotes in quotation marks "like this" to indicate direct quotes from your manuscript), or elaborate on this item by providing additional information not in the ms, or briefly explain why the item is not applicable/relevant for your study

Yes, this information is presented in Table 1 of the paper.

15-i) Report demographics associated with digital divide issues

In ehealth trials it is particularly important to report demographics associated with digital divide issues, such as age, education, gender, social-economic status, computer/Internet/ehealth literacy of the participants, if known.

	1	2	3	4	5	
subitem not at all important	○	○	○	○	◉	essential

Does your paper address subitem 15-i? *

Copy and paste relevant sections from the manuscript (include quotes in quotation marks "like this" to indicate direct quotes from your manuscript), or elaborate on this item by providing additional information not in the ms, or briefly explain why the item is not applicable/relevant for your study

Yes, this information is presented in Table 1 of the paper.

Você está editando sua resposta. O compartilhamento deste URL permitirá que outras pessoas também a editem.

INSERIR UMA NOVA RESPOSTA

16) For each group, number of participants (denominator) included in each analysis and whether the analysis was by original assigned groups

16-i) Report multiple “denominators” and provide definitions

Report multiple “denominators” and provide definitions: Report N’s (and effect sizes) “across a range of study participation [and use] thresholds” [1], e.g., N exposed, N consented, N used more than x times, N used more than y weeks, N participants “used” the intervention/comparator at specific pre-defined time points of interest (in absolute and relative numbers per group). Always clearly define “use” of the intervention.

	1	2	3	4	5	
subitem not at all important	○	○	○	○	●	essential

Does your paper address subitem 16-i? *

Copy and paste relevant sections from the manuscript (include quotes in quotation marks "like this" to indicate direct quotes from your manuscript), or elaborate on this item by providing additional information not in the ms, or briefly explain why the item is not applicable/relevant for your study

"IG and CG participants who completed the study (per-protocol analysis) achieved efficacy rates of 64.7% (CI 51.6%-77.8%) and 40.0% (CI 25.7%-54.3%), respectively (P=.02; OR 2.75, CI 1.20-6.29) and a number needed to treat of 4 (CI 2.0-19.0). In the intention-to-treat analysis, IG and CG participants achieved efficacy rates of 27.5% (CI 19.5%-35.5%) and 15.0% (CI 8.6%-21.4%), respectively (P=.02; OR 2.15, CI 1.13-4.08) and a number needed to treat of 8 (CI 4.0-43.0). Figure 3 shows the efficacy evolution during the 1-year follow-up in each study group according to both analytic approaches."

16-ii) Primary analysis should be intent-to-treat

Primary analysis should be intent-to-treat, secondary analyses could include comparing only “users”, with the appropriate caveats that this is no longer a randomized sample (see 18-i).

	1	2	3	4	5	
subitem not at all important	○	○	○	○	●	essential

Você está editando sua resposta. O compartilhamento deste URL permitirá que outras pessoas também a editem. INSERIR UMA NOVA RESPOSTA

Does your paper address subitem 16-ii?

Copy and paste relevant sections from the manuscript (include quotes in quotation marks "like this" to indicate direct quotes from your manuscript), or elaborate on this item by providing additional information not in the ms, or briefly explain why the item is not applicable/relevant for your study

Yes, see answer 16-i.

17a) For each primary and secondary outcome, results for each group, and the estimated effect size and its precision (such as 95% confidence interval)

Does your paper address CONSORT subitem 17a? *

Copy and paste relevant sections from the manuscript (include quotes in quotation marks "like this" to indicate direct quotes from your manuscript), or elaborate on this item by providing additional information not in the ms, or briefly explain why the item is not applicable/relevant for your study

See answer in 16-1). Besides: "BMI variances were similar for both groups. In the IG and CG, the mean BMI variances were 1.01 (CI 0.45-1.57) and 1.10 (CI 0.72-1.48), respectively, at 6 months and were 1.47 (CI 0.90-2.03) and 1.22 (CI 0.67-1.75), respectively, at 12 months. The between-group mean BMI differences at 6 and 12 months were 0.09 (CI 0.77 to 0.60; P=.80) and 0.25 (CI 0.53 to 1.03; P=.52), respectively."; Besides, check Table 2 for other summarized results.

17a-i) Presentation of process outcomes such as metrics of use and intensity of use

In addition to primary/secondary (clinical) outcomes, the presentation of process outcomes such as metrics of use and intensity of use (dose, exposure) and their operational definitions is critical. This does not only refer to metrics of attrition (13-b) (often a binary variable), but also to more continuous exposure metrics such as “average session length”. These must be accompanied by a technical description how a metric like a “session” is defined (e.g., timeout after idle time) [1] (report under item 6a).

	1	2	3	4	5	
subitem not at all important	○	○	○	○	◉	essential

Você está editando sua resposta. O compartilhamento deste URL permitirá que outras pessoas também a editem.

INSERIR UMA NOVA RESPOSTA

Does your paper address subitem 17a-i?

Copy and paste relevant sections from the manuscript (include quotes in quotation marks "like this" to indicate direct quotes from your manuscript), or elaborate on this item by providing additional information not in the ms, or briefly explain why the item is not applicable/relevant for your study

"A total of 17,111 messages and 2617 ratings were provided in the study, and there were 2311 ratings during the 12 months of follow up, 261 in the presmoking cessation phase, and 45 at 13 months or later, which were not part of this analysis." Besides, see also Multimedia Appendix 2, Figure 6, and Table 6.

17b) For binary outcomes, presentation of both absolute and relative effect sizes is recommended

Does your paper address CONSORT subitem 17b? *

Copy and paste relevant sections from the manuscript (include quotes in quotation marks "like this" to indicate direct quotes from your manuscript), or elaborate on this item by providing additional information not in the ms, or briefly explain why the item is not applicable/relevant for your study

Efficacy is a binary variable: "IG and CG participants who completed the study (per-protocol analysis) achieved efficacy rates of 64.7% (CI 51.6%-77.8%) and 40.0% (CI 25.7%-54.3%), respectively (P=.02; OR 2.75, CI 1.20-6.29) and a number needed to treat of 4 (CI 2.0-19.0). In the intention-to-treat analysis, IG and CG participants achieved efficacy rates of 27.5% (CI 19.5%-35.5%) and 15.0% (CI 8.6%-21.4%), respectively (P=.02; OR 2.15, CI 1.13-4.08) and a number needed to treat of 8 (CI 4.0-43.0). Figure 3 shows the efficacy evolution during the 1-year follow-up in each study group according to both analytic approaches."

18) Results of any other analyses performed, including subgroup analyses and adjusted analyses, distinguishing pre-specified from exploratory

Does your paper address CONSORT subitem 18? *

Copy and paste relevant sections from the manuscript (include quotes in quotation marks "like this" to indicate direct quotes from your manuscript), or elaborate on this item by providing additional information not in the ms, or briefly explain why the item is not applicable/relevant for your study

Yes, the paper includes Safety analysis (Adverse Events), Lost Data Pattern Analysis (check

Você está editando sua resposta. O compartilhamento deste URL permitirá que outras pessoas também a editem. INSERIR UMA NOVA RESPOSTA

18-i) Subgroup analysis of comparing only users

A subgroup analysis of comparing only users is not uncommon in ehealth trials, but if done, it must be stressed that this is a self-selected sample and no longer an unbiased sample from a randomized trial (see 16-iii).

	1	2	3	4	5	
subitem not at all important	(●)	( )	( )	( )	( )	essential

Does your paper address subitem 18-i?

Copy and paste relevant sections from the manuscript (include quotes in quotation marks "like this" to indicate direct quotes from your manuscript), or elaborate on this item by providing additional information not in the ms, or briefly explain why the item is not applicable/relevant for your study

This analysis was not performed in this work.

19) All important harms or unintended effects in each group

(for specific guidance see CONSORT for harms)

Does your paper address CONSORT subitem 19? *

Copy and paste relevant sections from the manuscript (include quotes in quotation marks "like this" to indicate direct quotes from your manuscript), or elaborate on this item by providing additional information not in the ms, or briefly explain why the item is not applicable/relevant for your study

"Safety: Adverse Events: Nineteen adverse events (11/19 [58%] associated with bupropion and 8/19 [42%] with varenicline) were identified in 14 IG participants (11.7%), whereas 23 adverse events (10/23 [43%] associated with bupropion and 13/23 [57%] with varenicline) were identified in 13 CG participants (10.8%) (P=.84). The most frequent events associated with bupropion were headache (6/21, 28.6%), insomnia (4/21, 19.0%), vertigo (4/21, 19.0%), acute abdominal pain (2/21, 9.5%), and others (5/21, 23.8%), and those associated with varenicline were nausea (6/21, 28.6%), acute abdominal pain (4/21, 19.0%), insomnia (3/21, 14.3%), vomiting (2/21, 9.5%), and others (6/21, 28.6%)."

Você está editando sua resposta. O compartilhamento deste URL permitirá que outras pessoas também a editem.

INSERIR UMA NOVA RESPOSTA

19-i) Include privacy breaches, technical problems

Include privacy breaches, technical problems. This does not only include physical “harm” to participants, but also incidents such as perceived or real privacy breaches [1], technical problems, and other unexpected/unintended incidents. “Unintended effects” also includes unintended positive effects [2].

	1	2	3	4	5	
subitem not at all important	○	◉	○	○	○	essential

Does your paper address subitem 19-i?

Copy and paste relevant sections from the manuscript (include quotes in quotation marks "like this" to indicate direct quotes from your manuscript), or elaborate on this item by providing additional information not in the ms, or briefly explain why the item is not applicable/relevant for your study

This paper doesn't deal with privacy issues of mHealth solutions.

19-ii) Include qualitative feedback from participants or observations from staff/researchers

Include qualitative feedback from participants or observations from staff/researchers, if available, on strengths and shortcomings of the application, especially if they point to unintended/unexpected effects or uses. This includes (if available) reasons for why people did or did not use the application as intended by the developers.

	1	2	3	4	5	
subitem not at all important	○	○	○	◉	○	essential

Does your paper address subitem 19-ii?

Copy and paste relevant sections from the manuscript (include quotes in quotation marks "like this" to indicate direct quotes from your manuscript), or elaborate on this item by providing additional information not in the ms, or briefly explain why the item is not applicable/relevant for your study

"Perceived Quality: Only 32 participants volunteered to respond to the questionnaire. However, not all participants who responded to the questionnaire completed it. The results are presented in Table 6."

Você está editando sua resposta. O compartilhamento deste URL permitirá que outras pessoas também a editem.

INSERIR UMA NOVA RESPOSTA

DISCUSSION

22) Interpretation consistent with results, balancing benefits and harms, and considering other relevant evidence

NPT: In addition, take into account the choice of the comparator, lack of or partial blinding, and unequal expertise of care providers or centers in each group

22-i) Restate study questions and summarize the answers suggested by the data, starting with primary outcomes and process outcomes (use)

Restate study questions and summarize the answers suggested by the data, starting with primary outcomes and process outcomes (use).

	1	2	3	4	5	
subitem not at all important	○	○	○	○	◉	essential

Does your paper address subitem 22-i? *

Copy and paste relevant sections from the manuscript (include quotes in quotation marks "like this" to indicate direct quotes from your manuscript), or elaborate on this item by providing additional information not in the ms, or briefly explain why the item is not applicable/relevant for your study

Yes, clinical outcomes and technical outcomes sub-sections deals mainly with these points.

22-ii) Highlight unanswered new questions, suggest future research

Highlight unanswered new questions, suggest future research.

	1	2	3	4	5	
subitem not at all important	○	○	○	◉	○	essential

Você está editando sua resposta. O compartilhamento deste URL permitirá que outras pessoas também a editem.

INSERIR UMA NOVA RESPOSTA

Does your paper address subitem 22-ii?

Copy and paste relevant sections from the manuscript (include quotes in quotation marks "like this" to indicate direct quotes from your manuscript), or elaborate on this item by providing additional information not in the ms, or briefly explain why the item is not applicable/relevant for your study

"These findings suggest the potential benefit of the intervention, as participants did not experience abnormal adverse effects and were more likely to be abstinent after 1 year. Nevertheless, further exploration of its efficacy is needed for validation in other cultures, and studies should include a larger sample size and real-world data."

20) Trial limitations, addressing sources of potential bias, imprecision, and, if relevant, multiplicity of analyses

20-i) Typical limitations in ehealth trials

Typical limitations in ehealth trials: Participants in ehealth trials are rarely blinded. Ehealth trials often look at a multiplicity of outcomes, increasing risk for a Type I error. Discuss biases due to non-use of the intervention/usability issues, biases through informed consent procedures, unexpected events.

	1	2	3	4	5	
subitem not at all important	○	○	○	○	◉	essential

Does your paper address subitem 20-i? *

Copy and paste relevant sections from the manuscript (include quotes in quotation marks "like this" to indicate direct quotes from your manuscript), or elaborate on this item by providing additional information not in the ms, or briefly explain why the item is not applicable/relevant for your study

Yes, there is a specific limitations sub-section dealing with this subitem.

21) Generalisability (external validity, applicability) of the trial findings

NPT: External validity of the trial findings according to the intervention, comparators, patients, and care providers or centers involved in the trial

Você está editando sua resposta. O compartilhamento deste URL permitirá que outras pessoas também a editem.

INSERIR UMA NOVA RESPOSTA

21-i) Generalizability to other populations

Generalizability to other populations: In particular, discuss generalizability to a general Internet population, outside of a RCT setting, and general patient population, including applicability of the study results for other organizations

	1	2	3	4	5	
subitem not at all important	○	○	○	◉	○	essential

Does your paper address subitem 21-i?

Copy and paste relevant sections from the manuscript (include quotes in quotation marks "like this" to indicate direct quotes from your manuscript), or elaborate on this item by providing additional information not in the ms, or briefly explain why the item is not applicable/relevant for your study

"Health care providers should consider incorporating this digital therapeutic solution in their usual care, as it can facilitate positive outcomes for participants willing to stop smoking."

21-ii) Discuss if there were elements in the RCT that would be different in a routine application setting

Discuss if there were elements in the RCT that would be different in a routine application setting (e.g., prompts/reminders, more human involvement, training sessions or other co-interventions) and what impact the omission of these elements could have on use, adoption, or outcomes if the intervention is applied outside of a RCT setting.

	1	2	3	4	5	
subitem not at all important	○	○	○	○	◉	essential

Does your paper address subitem 21-ii?

Copy and paste relevant sections from the manuscript (include quotes in quotation marks "like this" to indicate direct quotes from your manuscript), or elaborate on this item by providing additional information not in the ms, or briefly explain why the item is not applicable/relevant for your study

The control group was designed according to the routine care at Smoking Cessation Unit of Virgen del Rocio University Hospitals. The only difference of the RCT with routine care is the use of the mHealth solution (obviously) and the efficacy assessment through biochemical

Você está editando sua resposta. O compartilhamento deste URL permitirá que outras pessoas também a editem.

INSERIR UMA NOVA RESPOSTA

OTHER INFORMATION

23) Registration number and name of trial registry

Does your paper address CONSORT subitem 23? *

Copy and paste relevant sections from the manuscript (include quotes in quotation marks "like this" to indicate direct quotes from your manuscript), or elaborate on this item by providing additional information not in the ms, or briefly explain why the item is not applicable/relevant for your study

ClinicalTrials.gov NCT03553173; https://clinicaltrials.gov/ct2/show/NCT03553173

24) Where the full trial protocol can be accessed, if available

Does your paper address CONSORT subitem 24? *

Cite a Multimedia Appendix, other reference, or copy and paste relevant sections from the manuscript (include quotes in quotation marks "like this" to indicate direct quotes from your manuscript), or elaborate on this item by providing additional information not in the ms, or briefly explain why the item is not applicable/relevant for your study

ClinicalTrials.gov NCT03553173; https://clinicaltrials.gov/ct2/show/NCT03553173

25) Sources of funding and other support (such as supply of drugs), role of funders

Does your paper address CONSORT subitem 25? *

Copy and paste relevant sections from the manuscript (include quotes in quotation marks "like this" to indicate direct quotes from your manuscript), or elaborate on this item by providing additional information not in the ms, or briefly explain why the item is not applicable/relevant for your study

This research was funded by the H2020 European Commission research and innovation program (grant agreement 681120) as part of the SmokeFreeBrain project [illegible]

Você está editando sua resposta. O compartilhamento deste URL permitirá que outras pessoas também a editem. INSERIR UMA NOVA RESPOSTA

X27) Conflicts of Interest (not a CONSORT item)

X27-i) State the relation of the study team towards the system being evaluated

In addition to the usual declaration of interests (financial or otherwise), also state the relation of the study team towards the system being evaluated, i.e., state if the authors/evaluators are distinct from or identical with the developers/sponsors of the intervention.

	1	2	3	4	5	
subitem not at all important	○	○	○	◉	○	essential

Does your paper address subitem X27-i?

Copy and paste relevant sections from the manuscript (include quotes in quotation marks "like this" to indicate direct quotes from your manuscript), or elaborate on this item by providing additional information not in the ms, or briefly explain why the item is not applicable/relevant for your study

Yes Conficts of Interest is duly stated in the Conflicts of Interest sub-section.

About the CONSORT EHEALTH checklist

As a result of using this checklist, did you make changes in your manuscript? *

- ○ yes, major changes
- ○ yes, minor changes
- ◉ no

What were the most important changes you made as a result of using this checklist?

Not applicable

Você está editando sua resposta. O compartilhamento deste URL permitirá que outras pessoas também a editem.

INSERIR UMA NOVA RESPOSTA

How much time did you spend on going through the checklist INCLUDING making changes in your manuscript *

6 Hours

As a result of using this checklist, do you think your manuscript has improved? *

- ( ) yes
- (•) no
- ( ) Outro:

Would you like to become involved in the CONSORT EHEALTH group?

This would involve for example becoming involved in participating in a workshop and writing an "Explanation and Elaboration" document

- ( ) yes
- (•) no
- ( ) Outro:

Any other comments or questions on CONSORT EHEALTH

I had to shorten very hard most of the answers since the system seems to not accept large responses. That was quite upsetting since it made me spend 2 more hours reviewing and shortening answers, please consider using a different method for reporting this questionnaire. Thanks.

Você está editando sua resposta. O compartilhamento deste URL permitirá que outras pessoas também a editem. INSERIR UMA NOVA RESPOSTA

STOP - Save this form as PDF before you click submit

To generate a record that you filled in this form, we recommend to generate a PDF of this page (on a Mac, simply select "print" and then select "print as PDF") before you submit it.

When you submit your (revised) paper to JMIR, please upload the PDF as supplementary file.

Don't worry if some text in the textboxes is cut off, as we still have the complete information in our database. Thank you!

Final step: Click submit !

Click submit so we have your answers in our database!

Enviar

Nunca envie senhas pelo Formulários Google.

Este conteúdo não foi criado nem aprovado pelo Google. Denunciar abuso - Termos de Serviço - Política de Privacidade

Google Formulários

Você está editando sua resposta. O compartilhamento deste URL permitirá que outras pessoas também a editem.

INSERIR UMA NOVA RESPOSTA
